# Adiposity and cancer: meta-analysis, mechanisms, and future perspectives

**DOI:** 10.1101/2024.02.16.24302944

**Published:** 2024-02-18

**Authors:** Eleanor L. Watts, Steven C. Moore, Marc J. Gunter, Nilanjan Chatterjee

**Affiliations:** 1 Division of Cancer Epidemiology and Genetics, National Cancer Institute, Shady Grove, MD, USA; 2 Department of Epidemiology and Biostatistics, School of Public Health, Imperial College London, London, UK; 3 Department of Biostatistics, Bloomberg School of Public Health, Johns Hopkins University, Baltimore, USA; 4 Department of Oncology, School of Medicine, Johns Hopkins University, Baltimore, USA

## Abstract

Obesity is a recognised risk factor for many cancers and with rising global prevalence, has become a leading cause of cancer. Here we summarise the current evidence from both population-based epidemiologic investigations and experimental studies on the role of obesity in cancer development. This review presents a new meta-analysis using data from 40 million individuals and reports positive associations with 19 cancer types. Utilising major new data from East Asia, the meta-analysis also shows that the strength of obesity and cancer associations varies regionally, with stronger relative risks for several cancers in East Asia. This review also presents current evidence on the mechanisms linking obesity and cancer and identifies promising future research directions. These include the use of new imaging data to circumvent the methodological issues involved with body mass index and the use of omics technologies to resolve biologic mechanisms with greater precision and clarity.

## 1. Introduction

**Figure 1:**
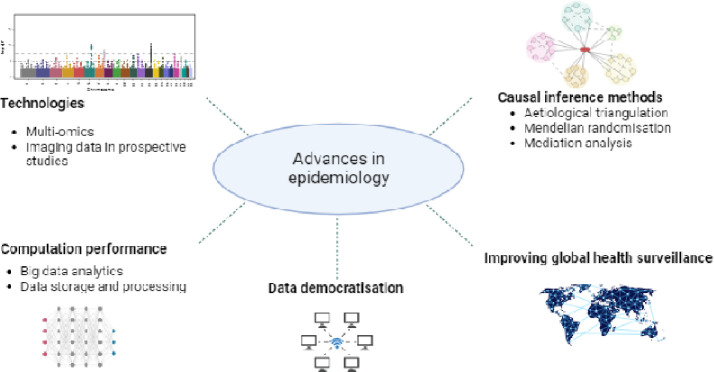
Recent transformative advances in epidemiological research

Epidemiologic studies first reported a link between elevated body mass index (BMI) and risk of cancer, specifically breast and endometrial cancers, in the 1960s and 1970s^1–3^. Since then, thousands of epidemiologic studies have examined obesity-cancer associations, and the underlying biology has been explored in experimental models. Recently, transformative advances in population health data and the generation of new molecular tools have expanded inquiries further—new healthcare database studies include millions of participants, Mendelian randomization (MR) studies offer insights towards causal inference, and omics studies enable the examination of thousands of biological analytes as candidate mechanistic mediators (Figure 1).

The pace of these research developments has made staying current on the literature challenging, even as the shifting obesity landscape has made it more urgent. For clinicians, newly approved, remarkably effective weight loss drugs (e.g. Ozempic/Wegovy) have generated overwhelming demand for obesity counselling, including assessments of obesity-related disease risks. Notably, only half of the population may recognise obesity as a cancer risk factor^4,5^. A current and comprehensive review on obesity and cancer is needed to help clinicians advise patients about their obesity-related risks of cancer. In providing a broad overview of current knowledge on obesity and cancer, such a review may also help researchers to target key research gaps more precisely.

In this review, we provide a comprehensive overview of the latest evidence linking obesity with cancer. In particular, we provide an up-to-date meta-analysis on the association of BMI with risk of the 25 most common cancers globally. This analysis evaluates data from 250 primary publications and up to 40 million participants, capturing up to 18 times more cancer cases compared with previous meta-analyses. We also review the latest evidence on potential mechanisms underlying the obesity-cancer link, incorporating results from new omics technologies, and discuss future advances in epidemiological research. Additionally, we discuss the clinical implications of the new obesity treatments and targeted screening in obese populations.

## 2. Background: Adiposity measurement and global trends

### 2.1. Adiposity measurement

**Figure 2:**
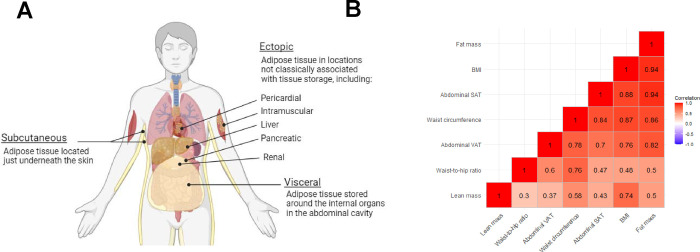
A. Distributions of adiposity storage B. Correlations of adiposity measures Partial Pearson correlations estimated in the UK Biobank population estimated by Christakoudi et al., 2021^9^. Lean mass and fat mass were measured using DXA, abdominal SAT and VAT were measured using MRI. Correlations were adjusted for age, weight change during the preceding visit, alcohol, physical activity, socioeconomic status, region (except for VAT and abdominal SAT) and for women, menopausal status and use of hormone replacement therapy. Body composition measurements were scaled by height, and then computed into sex-specific z-scores. Correlations were estimated separately for men and women, the median value by sex is displayed here. The largest difference in the correlation coefficient between sexes=0.23 (waist-to-hip ratio and fat mass, r=0.61 and 0.38 for men and women, respectively). Median difference in r=0.04. Abbreviations: BMI=body mass index, DXA=dual-energy x-ray absorptiometry, MRI=magnetic resonance imaging, SAT=subcutaneous adipose tissue, VAT=visceral adipose tissue.

The gold standard methods to measure fat are computed tomography, magnetic resonance imaging, and, to a lesser extent, dual-energy x-ray absorptiometry. These methods make it possible to measure total fat and fat in specific depots, such as subcutaneous adipose tissue (fat stored beneath the skin), and visceral adipose tissue (VAT—fat stored in the abdominal cavity), the latter of which is implicated in poor metabolic health^6^ (Figure 2A). Until recently, such gold standard measures have been too costly and complex to use in large-scale research studies. Instead, most clinical and research studies assess adiposity by BMI, calculated by dividing weight in kilograms by the square of height in metres. Participants are typically classified as normal weight (BMI of 18.5–24.9), overweight (25–29.9), and obese (30+) based on World Health Organization criteria^7^. BMI is often the subject of criticism because it does not differentiate fat mass from lean mass and, in fact, correlates highly with lean mass (r~0.7, see Figure 2B)^8–10^. Nevertheless, at the population level, BMI is more strongly correlated with body fat percentage (r~0.9) and other fat measures, thus its continued use in health research.

Waist circumference is also sometimes used as a proxy for fat distribution (e.g. abdominal fat or VAT). However, it is uncertain whether waist circumference measures fat distribution effectively^11–13^. Waist circumference measures VAT only marginally better than BMI and correlates with subcutaneous adipose tissue about as well as VAT, suggesting a lack of specificity (Figure 2B)^9,14^.

### 2.2. Global trends in obesity and obesity-related cancers

**Figure 3:**
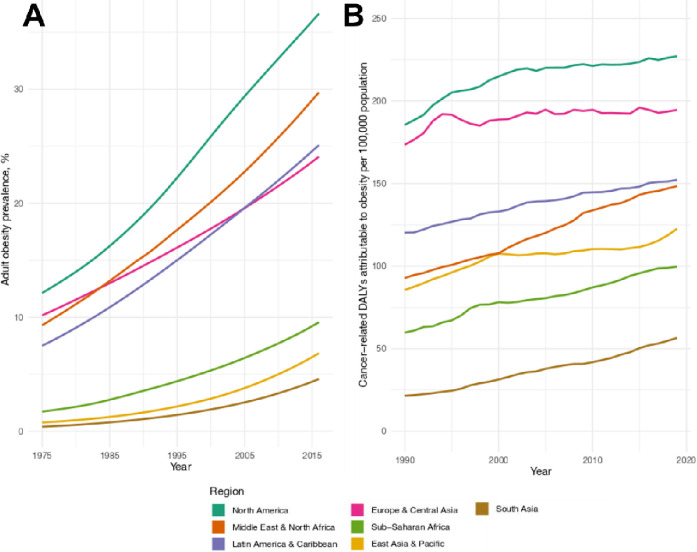
A: Global trends in obesity prevalence in adults 1975–2016. Based on obesity prevalence estimates (https://www.ncdrisc.org/) and the UN adult population estimates (https://population.un.org/). B: Age-adjusted rate of DALYs due to cancer attributable to high BMI, per 100,000 population. Based on the GBD Study data (https://www.healthdata.org/data-visualization/gbd-results). One DALY is equivalent to one lost year of full health. Abbreviations: BMI=body mass index; DALY=disability-adjusted life year; GBD=Global Burden of Disease; UN=United Nations

The obesity epidemic began in the 1970–80s. Originally localised to high income nations, it has subsequently evolved into a global phenomenon^15^. Currently, 12% of adult males and 16% of adult females worldwide are classified as obese^16^. In children, obesity has increased from 0.7% to 5.6% in girls and 0.9% to 7.8% in boys^16^. Among women, the highest prevalence of obesity is in Central Asia, the Middle East, and North Africa (age-standardised prevalence 35%). Among men, prevalence is highest in high income Western countries (30%)^16^. South Asia is experiencing the fastest relative increase in obesity rates, with prevalence increasing from 0.4% to 4.6% (Figure 3A). For children and adolescents, rates are rising fastest in low- and middle-income regions, whilst rates have plateaued in high-income regions^16^.

The pattern of weight gain over a lifetime also varies geographically. Obese adults in Western countries have, as a group, experienced longer cumulative exposure to high adiposity than obese adults in South and Southeast Asia, whose weight gain was more recent and intense. People of South and Southeast Asian ancestry may also have a greater tendency toward central adiposity than those of European ancestry^17,18^, possibly related to weight gain patterns and other environmental, and perhaps genetic, causes^19–22^. Access to weight loss treatments also varies by country, with lower access in low resource settings and for individuals with low socioeconomic status^23,24^.

Approximately 3.6% of all new cancer cases are estimated to be attributable to high BMI, this percentage is greater for women than for men (5.4% and 1.9%, respectively)^25^. High BMI is the third leading risk factor for the global cancer burden, behind tobacco and alcohol, accounting for 4.7 and 4.3% of disability-adjusted life years from cancer, for women and men, respectively^26^. This burden is highest in North America and has increased over time, but the fastest increases are occurring in low- and middle-income countries (Figure 3B). Greater obesity prevalence in younger populations might also be contributing to rising cancer diagnoses at earlier ages^27^.

## 3. Adiposity and cancer risk

### 3.1. Meta-analysis of BMI and cancer

Obesity is one of the most widely investigated risk factors in cancer epidemiology with early reports dating back to the 1960s and the results summarised by the World Cancer Research Fund (WCRF) via the Continuous Update Project (CUP) and the International Agency for Research on Cancer^28,29^. Nevertheless, for several reasons, a comprehensive new meta-analysis is urgently needed. First, although the WCRF reviews quantified the relationship between BMI and cancer, summary risk estimates were based on studies published years ago, from before 2008 to 2016 (depending on the cancer site). This precedes the publications from several major healthcare databases such as the National Health Insurance Service of Korea (N=~20 million)^30^, the Clinical Practice Research Datalink (N=~5 million)^31^, and the Information System for Research in Primary Care (N=~4 million)^32^ and comprehensive prospective cohorts (e.g., UK Biobank). Second, several common cancers were not previously evaluated in the WCRF reviews (thyroid, blood, and central nervous system cancers). Third, for smoking-related cancers, there is serious potential for residual confounding by smoking, regardless of any adjustment procedure used^33^. Meta-analyses specifically in never-smokers are needed to eliminate this concern. Fourth, these reviews predate the widespread adoption of MR, which can offer complementary evidence towards causal effects^34^. Lastly and most importantly, newly available data from East Asian studies (e.g. China Kadoorie Biobank, and the Asia Cohort Consortium) permit robust assessment of generalisability, which has received only modest previous coverage but could reveal intriguing and important facets of the BMI-cancer association. To address these limitations, we conducted an updated meta-analysis of the associations between BMI and risk of common cancers.

#### 3.1.2. Literature search

We meta-analysed results for the 25 most common cancers by global incidence. We first collated results of prospective studies in prior WCRF meta-analyses^29^. For cancer types not covered by WCRF (thyroid cancer, leukaemia, non-Hodgkin lymphoma (NHL), glioma and meningioma), we used the latest applicable meta-analysis^35–37^. We then built on prior meta-analyses through a PubMed literature search starting the year of the last review for each type of cancer (terms and protocol in Supplementary Methods). In brief, eligible studies met the following criteria: 1) prospective risk estimates of BMI-cancer associations; 2) ≥50,000 participants (or a more recent estimate from a study in the last review); 3) continuous risk estimates or ≥3 categories of BMI; 4) estimates for subtype-specific cancers (where needed); 5) risk estimates for never-smokers (smoking-related cancers only); 6) adjustment for smoking or evaluation of confounding by smoking; 7) models did adjust for early-life BMI or other measure of adiposity; 8) not duplicative (risk estimates with the greatest number of cases were extracted where duplicate study populations were identified). More information regarding cancer type definitions, literature search, data collection and risk estimate harmonisation are available in Supplementary Methods. Extracted data and results are publicly available for use by the scientific community (Supplementary Data).

#### 3.1.3. Statistical analysis

Pooled estimates were estimated using random effects meta-analysis and heterogeneity between studies was assessed using I^2^, tau^2^ and the 25^th^ and 75^th^ percentile (interquartile range (IQR)) of the relative risks (RR) for each cancer site (Supplementary Methods). In sensitivity analyses, we examined the effect of removing risk estimates from large administrative health databases (Supplementary Methods). These results are described in 3.1.4 Main Results below. The generalisability of BMI and cancer associations across regions is described in detail in its own section (3.1.5 Generalisability). We assessed heterogeneity by Cochran’s Q, focusing on the following geographic regions: Australia, East Asia, South Asia, West Asia, Europe, and North America.

#### 3.1.4. Main results

We reviewed 2,425 articles, of which 250 met our inclusion criteria. From these, we extracted 688 BMI-cancer associations (some studies evaluated multiple cancer types). The cohorts originated from 25 countries and six major geographic regions (Figure 4A), with more recent cohorts having far greater sample sizes and numbers of incident cancer cases than cohorts before ~2014 (Figure 4B). This is primarily driven by publications from large health databases such as the National Health Insurance Service of Korea, which recorded >120,000 incident colorectal cancers alone. Consequently, participant numbers were far larger in the current meta-analysis than in preceding meta-analyses. For example, the colorectal cancer analysis had 40 million participants as compared with 4.8 million in the prior WCRF review. The number of cancer cases increased substantially as well—there was a doubling in cases for all types of cancer and up to 18 times more for some types (Supplementary Table 3).

In aggregate, this review includes 988,835 incident cancers across the 25 types of cancer. The smallest case count was for oesophageal squamous cell cancer (SQ, N=893; never-smokers only) and the largest for colorectal cancer (N=270,000) (Supplementary Figure 1, Supplementary Table 1). Cancer cases originated from Europe (47.8%), East Asia (40.0%), and North America (11.6%) (Supplementary Figure 2A), with some variability by cancer type (e.g., there were no studies of gastric non-cardia cancers in North America, Supplementary Figure 2B). Details for each study including model adjustments, N cases, N analytic population, risk estimates, follow-up duration and originally reported units are available from Supplementary Data 1.

**Figure 5:**
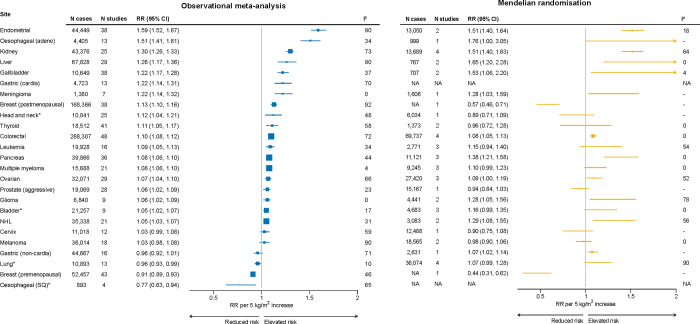
Associations between BMI with cancer risk, RR per 5 kg/m^2^ increase in BMI RRs are represented by squares (with their 95% CIs as lines). Observational risk estimates were calculated using random effects meta-analysis. Heterogeneity is quantified using I^2^, an I^2^ close to 100% indicates substantial heterogeneity but can be affected by the number of studies and the precision of individual study estimates. Further details of model adjustments, follo time, analytic population for each study are available from Supplementary Data 1. For Mendelian randomisation studies, results were selected from single genetic ancestry populati reduce confounding by population structure and the I^2^ quantifies heterogeneity between studies rather than between individual SNPs. References for the Mendelian randomisation studies follows: oesophageal (adenocarcinoma)^44^, meningioma^45^, breast (pre and postmenopausal)^46^, head and neck^47^, prostate^48^, glioma^49^, gastric (non-cardia)^50^, and for other cancers^51^. *Observational risk estimates based on never-smokers only. Abbreviations: Adeno=adenocarcinoma, BMI=body mass index, CI=confidence interval, NHL=non-Hodgkin lymphoma, RR=risk ratio, SNP=single nucleotide polymorphism.

Higher BMI was associated with increased risks of endometrial cancer (RR=1.59 per 5 kg/m^2^ increase, 95% CI 1.52–1.67), oesophageal adenocarcinoma (1.51, 1.41–1.61), kidney (1.30, 1.26–1.33), liver (1.26, 1.17–1.36), gastric (cardia) (1.22, 1.14–1.31), gallbladder (1.22, 1.17–1.28), meningioma (1.22, 1.14–1.32), breast (postmenopausal) (1.13, 1.10–1.16), thyroid (1.11, 1.05–1.17), colorectal (1.10, 1.08–1.12), leukaemia (1.09, 1.05–1.13), pancreatic (1.08, 1.06–1.10), multiple myeloma (1.08, 1.05–1.10), ovarian (1.07, 1.04–1.10), prostate (aggressive) (1.06, 1.02–1.09), and NHL (1.05, 1.03–1.07). BMI was inversely associated with breast (premenopausal) (0.91, 0.89–0.93) (Figure 5A). For cancers where smoking is a strong risk factor, we observed increased risks for head and neck (1.12, 1.04–1.21) and bladder cancer (1.05, 1.02–1.07), and decreased risks for oesophageal SQ (0.77, 0.63–0.94) and lung cancer (0.96, 0.93–0.99) for never-smokers. Removing the large healthcare database studies did not materially affect results (% change in RRs <5% for all sites), even though many cases were eliminated for some cancers (Supplementary Table 4). Individual study estimates for each site are available from Supplementary Figures 3–27.

Despite the considerable expansion in sample size, risk estimates were remarkably consistent (though more precise) compared with those from previous reviews (median absolute change in RRs=0.03, Supplementary Table 3). This suggests that, for many types of cancer, BMI-cancer associations have reached stability, rendering further data collection unnecessary. The lack of change in RRs was surprising as our analysis included substantial new data from East Asia, whose RRs were expected to differ from those obtained in North America and Europe. There were, however, important key differences that we discuss further in section 3.1.5. Generalisability below.

RRs were consistent in direction across studies, though with some quantitative heterogeneity (i.e., I^2^> 0%). Heterogeneity was largest for endometrial and liver cancers and melanoma (I^2^ of 90% for each). For endometrial cancer, RRs were consistently high but with varying magnitudes (IQR of RRs across studies:1.49–1.77). The association of BMI with endometrial cancer varies by menopausal hormone therapy use and differences in hormone use by population could possibly explain heterogeneity. For liver cancer, the range in associations was especially large (IQR of RRs: 1.03–1.53), which could relate to differences in aetiology (in Europe and North America non-alcoholic fatty liver disease is the main cause of liver cancer while in East Asia the prevalence of hepatitis B is much higher^38,39^), and possible non-linear associations^31,32^. For melanoma, results appeared far more positive in Scandinavian countries with generally null associations elsewhere.

Our analysis of never-smoking participants enabled us to examine BMI’s association with risk of smoking-related cancers, without confounding by smoking. The results suggest that an elevated BMI is a risk factor for head and neck cancer and possibly protective for oesophageal SQ. There were also statistically significant associations for lung and bladder cancers, though RRs were nearly null and not likely clinically significant. These results constitute distinctive contributions to the literature as prior studies generally had few cases of smoking-related cancers among never-smokers, particularly bladder and oesophageal SQ cancers (N=0 for never-smoking bladder cancer; N=100 for never-smoking oesophageal SQ)^40,41^. It is plausible that the associations of BMI with smoking-related cancers may be different for smokers, but this is difficult to evaluate in observational studies. Future MR analyses stratified by smoking status might increase our understanding of these relationships.

The findings from MR studies broadly align with the observational risk estimates (though with less precision), except for female breast cancer, where higher genetically predicted BMI appears to be protective of postmenopausal breast cancer (Figure 5B). This may be due to the protective role of early-life BMI on postmenopausal breast cancer^42^, suggesting that observational positive associations with postmenopausal breast cancer are likely driven by adult weight gain^43^.

#### 3.1.5. Generalisability

A key aim of our analysis was to explore the generalisability of BMI and cancer associations by geographic region. Consistent associations across regions support causality, indicating universality rather than regional confounding patterns^34^. Regional variations could reflect differences in confounding, but also could reveal facets of disease biology. The strength of BMI’s association with certain cancers reliably depends on the presence or absence of certain cofactors, such as menopausal hormone therapy (see Interaction of obesity section below). Understanding regional variations can provide clues about these cofactors and biological pathways. Quantifying the relative risks relevant by region is also essential for accurately estimating obesity’s impact on cancer.

Despite hundreds of prospective epidemiological studies on obesity and cancer, the published literature is notably restricted in its geographic scope. Our meta-analysis revealed a lack of prospective studies on BMI and cancer risk from Africa, South or Central America, Eastern Europe, South or Central Asia, the Caribbean, and the Pacific Islands (Figure 4A). Notably, the countries included in our meta-analysis of BMI and cancer risk constitutes just 30% of the global population^52^.

**Figure 6:**
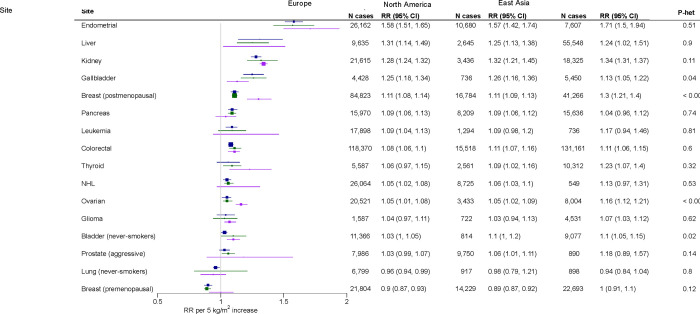
Associations between BMI and cancer risk by region Figure restricted to sites with >500 cases per region and estimates available for Europe, North America, and East Asia. Risk estimates were calculated using random effects meta-analysis. P-heterogeneity was estimated using the Q-statistic and includes the regions Australia, South Asia, and West Asia (not shown here due to the low number of cancers). Full results for each site and region are available from Supplementary Figures 28–52. Abbreviations: CI=confidence interval, NHL=non-Hodgkin lymphoma, RR=risk ratio, SD=standard deviation, SQ=squamous cell carcinoma

Suggestive of important population-specific variation in the obesity-cancer link, the association of BMI with risk of female reproductive cancers (postmenopausal breast and ovarian), bladder, and gallbladder cancers differed notably by region (Figure 6). For postmenopausal breast cancer, the difference was especially striking. The RR per 5 kg/m^2^ increment was 1.11 in North America and Europe but 1.30 in East Asia—a near tripling of the excess risk. For ovarian cancer, the RR was also higher in East Asia (RR=1.16) than in North America and Europe (RR=1.05 for both). For bladder cancer, the BMI association was modestly weaker in Europe than elsewhere and for gallbladder cancer, the BMI association was weaker in East Asia.

The heterogeneity of associations by region may stem from differences in background risk factors. For instance, postmenopausal hormone therapy is not widely used in China^53^ and the association of BMI with breast cancer risk is known to be stronger among non-hormone-users^54^. Additionally, postmenopausal oestrogen levels, which are associated with breast and ovarian cancer, may be on average lower in East Asian women than European or US women^55–57^, and so a high BMI could contribute more, on a relative basis, to oestrogen levels in East Asia. For gallbladder cancer, the association with BMI was notably weak in South Korea, where liver fluke may perhaps be more important as a risk factor^58^. Differences in fat distribution between Europeans/North Americans and East Asians do not likely explain heterogeneity since such differences would have to explain both stronger *and* weaker associations observed in East Asians.

From a public health perspective, the results for breast cancer are particularly important. Current estimates of the cases of breast cancer attributable to obesity in East Asia are based on RRs from Europe and North America^26^. Our results indicate that these estimates may understate the cases attributable to obesity by a factor of two or more. Future studies should use region-specific estimates whenever possible.

### 3.2. Waist circumference vs. BMI and cancer risk

#### 3.2.1. Background

Higher waist circumference has been suggested to increase risks of some cancers, with a greater magnitude of effect than BMI. However, studies have generally been based on a small number of cancer sites or have not directly compared measurements. To address this, we aimed to systematically compare associations of waist circumference and BMI with cancer risk.

#### 3.2.2. Study selection and statistical analysis

We examined the studies identified in our meta-analysis outlined above which met the following criteria: 1) reported results for both BMI and waist circumference using the same models and populations; 2) ≥5 types of cancers investigated, to reduce the possibility of publication bias; 3) results not mutually adjusted (due to collinearity of measurements)^12^; 4) results reported per SD or where risk estimates could be converted to SDs. The meta-analysis was restricted to studies that reported on both BMI and waist circumference to minimise random noise and protect against potential publication bias. Heterogeneity by adiposity measurement was assessed using the Wald statistic (Supplementary Methods).

#### 3.2.3. Results

**Figure 7:**
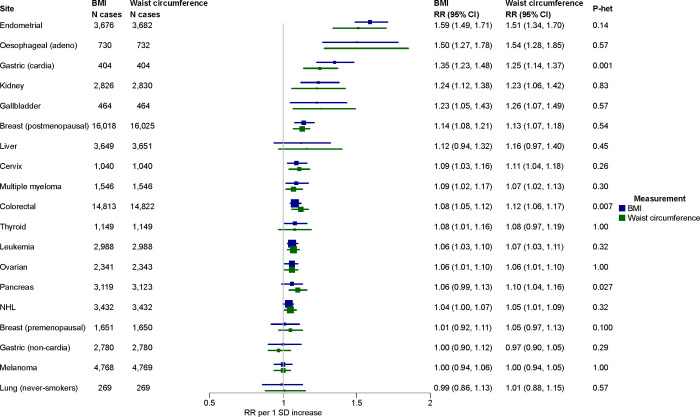
Associations of BMI, waist circumference and cancer risk, per 1 SD increase Associations estimated from 6 studies^32,59–63^, pooled using random effects meta-analysis. Heterogeneity in the associations with each cancer site by adiposity measure was estimated using the Wald statistic. Abbreviations: CI=confidence interval, NHL=non-Hodgkin lymphoma, RR=risk ratio, SD=standard deviation

In total we identified 6 studies from up to 1.44 million participants, comprising 16,000 cases from four countries (China, Spain, UK, USA)^32,59–63^. Risk associations for each study and type of cancer are available from Supplementary Figures 53–76. Risk associations were significantly different for gastric cardia (RR per 1 SD increase in BMI:1.35, 1.23–1.48 and waist circumference: 1.25, 1.14–1.37), colorectal (1.08, 1.05–1.12 and 1.12, 1.06–1.17), and pancreatic cancers (1.06, 0.99–1.13 and 1.10, 1.04–1.16) (Figure 7). There is also some evidence from MR studies to support slightly larger associations with abdominal adiposity for pancreatic and colorectal cancers^64,65^. For smoking-related cancers, we also observed consistently larger differences in associations when populations were not restricted to never-smokers (e.g., lung cancer BMI: 0.91, 0.85–0.98 and waist circumference: 1.01, 0.94–1.08; P-het<0.001, Supplementary Figure 73), which may relate to the dual effects of smoking on fat distribution and appetite suppression^66^.

Overall, although we observed some differences in the associations of BMI and waist circumference particularly for the digestive cancers, the aggregate difference in the risk ratios was small (median absolute difference in RRs=0.02).

### 3.3. Adiposity over the life course

Cancer develops due to an accumulation of mutational events over the life course and many cancers also have long latency periods before becoming clinically apparent. Therefore, it is plausible that adiposity could predominantly affect risk of some cancer sites earlier in life, with diminished importance later in life, regardless of weight change. The evidence from observational studies suggests that early life obesity may be a major risk factor for pancreatic cancer, while adult obesity is more important for endometrial and kidney cancer^67^. In MR studies, early-life body size and adult BMI are both associated with obesity-related cancers, but relationships for early-life BMI generally attenuate after accounting for adult BMI in multivariable models^68,69^, with the exception of breast cancer^70^. However, a limitation of studies examining adiposity over the life course is the inherent interdependence of childhood BMI, gain in BMI during adulthood, and adult BMI. These measures are not algebraically separable (i.e., childhood BMI + gain in BMI=adult BMI), therefore the distinct role of each in cancer risk cannot be fully disentangled in statistical models.

### 3.4. Intentional weight loss and cancer

Evidence for the role of intentional weight loss on cancer risk is relatively sparse. Bariatric surgery is a highly effective weight loss intervention (e.g. 31% reduction in body weight (95% CI, 30%–32%) in 1-year)^71^. This surgery has been linked to reduced risk of cancer in women, particularly for endometrial and possibly postmenopausal breast cancers (e.g. Odds Ratio=0.43, 95% CI 0.26–0.71 and 0.49, 95% CI 0.33–0.72, respectively)^72–74^. For other cancer sites the associations are less clear^72^. Similarly, in prospective cohort studies, associations of weight loss with breast and endometrial cancers appear to be the most robust (for instance, Hazard Ratio=0.68, 95% CI 0.50–0.93 for postmenopausal breast cancer women who sustained ≥9 kg weight loss)^75^. Evidence from randomised controlled trials of dietary interventions remains inconclusive due to low statistical power and limited follow-up duration^76^. A significant challenge in investigating intentional weight loss and cancer risk is the potential for concurrent lifestyle modifications (such as increased physical activity, healthier diet, and reduced smoking).

### 3.5. Cancer disparities and role of adiposity

In high income nations obesity is more prevalent among socially disadvantaged groups, including ethnic/racial minorities and individuals from lower socioeconomic backgrounds^77^. This may contribute to disparities in cancer risk. In the US, the prevalence of obesity in Black women (56.2%) is much higher than in White women (39.0%)^78^, for instance. Largely because of this, the proportion of breast cancers attributable to obesity is substantially higher in Black women (28.3%) than White women (15.4%)^79^. A high prevalence of obesity and diabetes may also explain some of the higher risk of pancreatic cancer in Black men and women^80^. For individuals in low socioeconomic groups, obesity and related risk behaviours contribute to higher risk of early onset colon cancer, especially for the right-sided colon^81^.

These data indicate that the higher prevalence of obesity in disadvantaged groups increases their burden of cancer, at least for the few cancers that have been examined to date. A more systematic examination of the cancer burden imposed by obesity in disadvantaged groups across all cancer types is needed.

## 4. Molecular mechanisms

Adipose tissue is a complex endocrine system that interacts with numerous organs to maintain metabolic homeostasis. Body fat levels correlate with circulating levels of 100s to 1000s of different metabolites, proteins, and other biomarkers^8,82–85^. It is possible that only a small fraction of these, e.g. 5% or fewer, are relevant to cancer risk^82^. Moreover, only some individuals with obesity may have elevated levels of the cancer-pertinent factors. Approximately 10–30% of individuals with obesity maintain metabolic health characterised by an absence of conditions such as gallstones, diabetes, hyperinsulinemia, dyslipidaemia, or fatty liver^6^. These individuals may have lower cancer risks compared with their metabolically unhealthy counterparts, though risk remains higher than those who are metabolically healthy and lean^6,86^.

A detailed mechanistic understanding of the obesity-cancer association is essential for developing well-informed public health and clinical interventions. The benefits of such a mechanistic understanding can be summarised by the acronym CRIB:

- Causality: Establishing a mechanistic understanding helps verify causality of the obesity-cancer association, thus providing strong justification for interventions.
- Risk stratification: Information on mechanisms can be used to stratify obese individuals for targeted interventions.
- Intervention refinement: Information on mechanisms can be used to optimise interventions so that they maximally reduce levels of these factors.
- Biology: Information on mechanisms may provide biological clues that facilitate development of targeted treatments (e.g. drugs).

Distinguishing between those obesity-related factors relevant to cancer and those that are not continues to be a major research challenge. A key tool for this is *mediation analysis* (Box 1), a method that allows many factors to be modelled simultaneously to determine those that quantitatively contribute the most to the obesity-cancer association.

### 4.1. Classical mediators

**Figure 8:**
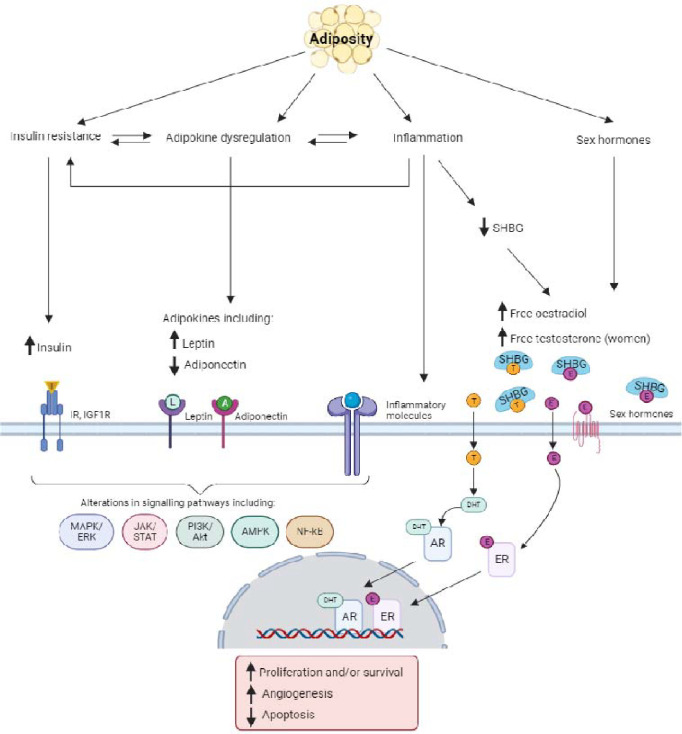
Simplified schematic of the established biological mechanisms between obesity and cancer Abbreviations: AMPK=AMP-activated protein kinase, AR=androgen receptor, DHT=dihydrotestosterone, E=oestradiol, ER=oestradiol receptor, ERK=extracellular signal-regulated kinase, IGF1R=insulin-like growth factor 1 receptor, IR=insulin receptor, JAK=Janus kinase, PI3K=phosphoinositide 3-kinase, MAPK=Mitogen activated protein kinase, NF-kB=Nuclear factor kappa B, SHBG=sex hormone-binding globulin, STAT=signal transducer and activator of transcription, T=testosterone. Created with BioRender.com

There are three classical hypotheses to explain the positive relationship between obesity and cancer risk: altered sex hormone concentrations, insulin resistance with hyperinsulinemia, and chronic inflammation (Figure 8).

#### 4.1.1. Sex steroid hormones

Adipose tissue is the major source of circulating oestrogen in men and postmenopausal women, facilitating the conversion of androgens to oestrogens^89,90^. Additionally, adipose tissue expresses 17 - hydroxysteroid dehydrogenase which converts less active forms of sex hormones into testosterone and oestradiol, which are more biologically potent^91^. The bioavailability of circulating testosterone and oestradiol is modulated by sex hormone-binding globulin (SHBG), which binds to these hormones, limiting their diffusion into target tissues^92^. Higher adiposity has a strong inverse correlation with SHBG concentration, relating to increased liver fat, insulin and proinflammatory cytokines^93^. For women, lower SHBG leads to higher circulating concentrations of free testosterone and free oestradiol^90,94^. While for men, lower SHBG leads to a compensatory decrease in testosterone production, keeping free testosterone constant, except for men with severe obesity^89^.

Higher oestradiol has been associated with an increased risk of breast (particularly ER+ tumours), endometrial and ovarian (particularly endometrioid and serous subtypes) cancers in women in both observational and MR analyses^90,94–97^. Higher free testosterone may also increase risks of ER+ breast and endometrial cancers^98^.

For postmenopausal women, pooled observational studies have suggested that the positive association between BMI and breast cancer may be largely driven by oestradiol^99^. For endometrial cancer, risk may increase when high levels of oestradiol are not counterbalanced by progesterone in the uterus^100^. In an observational analysis, 21% of the association between BMI and endometrial cancer was estimated to be mediated by oestradiol^101^. While in MR mediation analysis, free testosterone and SHBG were estimated to mediate 15% and 7% of the relationship between BMI and endometrial cancer risk, respectively, though oestradiol and progesterone were not included in this analysis^102^.

For prostate cancer, men with severe obesity have low free testosterone levels, whereas free testosterone may be positively associated with prostate cancer risk^103^. However, there is some observational evidence that very low free testosterone levels may increase risks of high-grade disease^104,105^.

#### 4.1.2. Hyperinsulinemia

Excess adiposity, particularly dysregulated visceral fat, induces insulin resistance and compensatory hyperinsulinemia^106^. Insulin is a growth factor that binds to intracellular insulin receptors with high affinity, stimulating the activation of signalling cascades which regulate metabolism and cell growth (Figure 8). High fasting insulin concentrations are associated with increased risks of breast^107,108^, endometrial^102^, pancreatic^109,110^, colorectal^111^, renal^112^, gastric^113,113^ and liver cancers^114,115^. In an MR mediation analysis, high fasting insulin mediated 19% (95% CI: 5–34%) of the association of obesity with endometrial cancer^102^, and observational studies have reported that high insulin may also largely mediate associations between BMI and colorectal cancer^116,117^.

Insulin-like growth factors (IGFs) are structurally similar to insulin and stimulate cell growth and inhibit apoptosis^118^. Although there is strong evidence to suggest that higher IGF-I is associated with an increased risk of breast, prostate and colorectal cancers^119–122^, individuals with obesity have lower circulating IGF-I concentrations in comparison with those with BMIs in the healthy range^123^. However, adipocytes produce IGF-I and other signalling molecules and so it is possible that excess adiposity may exert local tumour-promoting effects via increased IGF-I signalling rather than systemically^124,125^.

#### 4.1.3. Inflammation

Obesity is considered a chronic low grade inflammatory state. Adipose tissue directly secretes adipokines, which regulate inflammation and other metabolic processes^126^. The most well-characterised adipokines are leptin and adiponectin. Leptin is hypothesised to be pro-inflammatory, while adiponectin is an anti-inflammatory and insulin-sensitising. While positive associations between leptin and obesity-related cancers have been observed^127–133^, the totality of the evidence for associations of leptin with cancer remains inconsistent^134,135^. MR analyses also do not support a direct role, although analyses may be underpowered to detect more modest associations^136^. Adiponectin is primarily secreted by VAT and is downregulated in obesity due to complex signalling relating to inflammation, oxidative stress and adipocyte hypertrophy^137^. In epidemiological studies, higher adiponectin is associated with a lower risk of colorectal cancer, but associations are generally attenuated after accounting for BMI^128,136,138,139^. Evidence for associations with other obesity-related cancers remains unclear^135,136,140–145^, and are not supported by MR studies^102,146^.

Other biomarkers of inflammation are candidate underlying mechanisms for obesity-related cancer, including C-reactive protein (CRP). In observational analyses, higher CRP is generally associated with increased risks of lung, colorectal and postmenopausal breast cancer^133,135,147,148^. However, MR analyses are not supportive of a causal relationship^102,135,136,149^.

Limitations of investigating these non-specific markers of inflammation include reverse causation from preclinical disease and difficulty in disentangling correlated biomarkers from each other and from other metabolic changes associated with obesity (e.g., the correlation of leptin with body fat % is r=0.85) and from shared signalling pathways^150^. Together these limitations may partially explain difficulties in identifying core mechanistic mediators. Recent MR analyses have suggested a range of inflammatory marker associations with cancer, appearing to be site and subtype specific^151,152^. Expanding the breadth of the integration of genetic and biomarker data to identify specific genetic instruments alongside the consideration of biological function may help to reduce the possibility of pleiotropy and identify the inflammatory agents driving the aetiological associations.

Adipose tissue can also have local effects on target tissue, which create a physiological milieu which might promote cancer development^153^. However, due to the complexity of investigating local tissues, the prospective associations of local inflammation with cancer remain poorly characterised.

### 4.2. “Omics”-identified biologic factors

In recent years, a proliferation of “omics” technologies—such as metabolomics and proteomics—has made it possible to examine hundreds to thousands of biological analytes simultaneously in biological specimens. This has allowed epidemiologists to expand beyond the “classical mediators” of the adiposity-cancer relationship and to instead examine factors such as the metabolism of carbohydrates, amino acids, and lipids. While “omics” data are still emerging, metabolomics studies have identified some strong candidate mediators of the obesity-cancer link. These studies have tended to take two approaches to studying mediation, namely: 1) evaluation of individual metabolites and 2) evaluation of adiposity “signatures”.

For example, in eight prospective studies^82,154–160^, 13 individual metabolites were identified that replicably associate with risk of postmenopausal breast cancer, most of which also correlate with BMI^82,161,162^. These include several understudied sex steroid hormone metabolites whose association with breast cancer risk was nevertheless independent from oestradiol and dehydroepiandrosterone (DHEA)-sulphate^82,155^, one of which (16α-hydroxy DHEA 3-sulphate) mediated 34% of the BMI-breast cancer relationship^82^. Other candidate mediators—i.e. metabolites associated with both BMI and breast cancer risk—included asparagine and phosphatidylcholines with 34 to 36 carbon atoms^155,157^. For kidney cancer, C3-DC-CH3 carnitine and C5 carnitine were estimated to mediate 20% of the BMI-kidney cancer association^163^ and suggestive evidence was found for several phosphatidylcholines^164^. Lastly, a large European study of eight cancer types (breast cancer, colorectal cancer, endometrial cancer, kidney cancer, gallbladder cancer, liver cancer, advanced prostate cancer and localised prostate cancer) found nine metabolites/metabolite clusters robustly associated with cancer risk, including the BMI-associated metabolites of 36-carbon phosphatidylcholines, glutamine, and butyrylcarnitine^165^.

In studies of adiposity signatures, a metabolite score predictive of BMI is obtained using multivariable methods like Least Absolute Shrinkage and Selection Operator (LASSO) and modelled in relation to cancer risk. This helps reduce data dimensionality and may lead to more powerful associations, but also assumes that associations stem from a general obesity metabolic phenotype rather than specific and/or sporadic metabolic states that only sometimes accompany obesity (such as when obesity leads to gallstones)^166^. In studies to date, adiposity signatures were not shown to substantially mediate the obesity-cancer associations for advanced prostate cancer^167^ or breast cancer^168^. There was 12% mediation for endometrial cancer^169^ and results were mixed for colorectal cancer^169,170^. This relative lack of mediation, when compared with studies of “classical mediators”, may reflect low statistical power or that adiposity signatures are not sufficiently specific measures of the key metabolites.

Going beyond metabolomics, some early proteomics studies have examined the association of dozens to thousands of proteins with risk of cancer^171–176^. While some intriguing associations have been identified, no studies have examined the role of the circulating proteome in mediating the obesity-cancer link beyond specific targeted proteins such as insulin or inflammatory factors.

### 4.3. Microbiome

The microbiome is dysregulated in obesity with an observed reduction in the diversity of gut bacteria and decreased bacterial heterogeneity, which has been linked to increased systemic inflammation and effects on specific host receptors such as Toll-like receptors (TLRs). Lipopolysaccharide (LPS), a component of the outer membrane of Gram-negative bacteria, can enter the circulation from the intestinal lumen via leaky intestinal tight junctions and can infiltrate tissues such as the liver or adipose tissues, triggering an innate immune response and increased pro-inflammatory cytokine expression^177,178^. Given that inflammation plays a role in the progression of many cancers, it is plausible that obesity-induced perturbations of the gut microbiota are a contributing factor in the link between adiposity and cancer. However, our understanding of the role of the gut microbiome in mediating the adiposity and cancer relationship is limited, as large-scale epidemiological studies have generally not collected pre-diagnostic stool samples from participants to measure gut microbiota composition. The gut microbiota are a source of numerous metabolites such as short chain fatty acids, steroid hormones and amino acids, many of which can induce physiological effects on host cells^179,180^. The specific role many of these metabolites play in cancer development and whether they contribute to pathways linking obesity with cancer is not well understood but remains a growing area of scientific interest.

### 4.4. Interaction of obesity with other cancer risk factors and genetic susceptibility

Multiplicative effects of obesity with environmental or genetic risk factors implies the risk difference associated with obesity is the strongest in the presence of these other factors. Identification of multiplicative effects could lead to an understanding of mechanisms of action and to identify subgroups of the population with specific profiles who may obtain the most benefit from modification of obesity or metabolic factors, such as targeted behavioural or chemoprevention (e.g., metformin, aspirin). Most notably for female reproductive cancers, the magnitude of the obesity association appears to be greater for women who are never users of hormone replacement therapy, providing further evidence that oestradiol lies on the causal pathway^181–183^. There is also evidence of a synergistic relationship between BMI, gastro-oesophageal reflux and oesophageal adenocarcinoma^184^.

There is currently limited evidence to support the presence of interactions between BMI and polygenic risk scores^185,186^. However, polygenic risk scores aggregate association over many genes, which are likely to affect risk of cancer through different pathways. Therefore, evidence of interactions may be washed away due to different patterns of interactions across pathways. Genome-wide GxE interaction scans (GWIS) are an alternative approach that can help identify biologically meaningful interactions, which may reveal novel tumorigenic pathways modified by environmental exposures (such as obesity or biomarkers of metabolic dysfunction). Newly developed methods, mainly within studies of colorectal cancer, have identified genetic variants whose effects are modified by environmental factors, some of which would have been missed through studying genetic effects alone. For instance, interactions have been observed between BMI and known colorectal cancer loci such as *SMAD7*^187^. A recent analysis in the Genetics and Epidemiology of Colorectal Cancer Consortium (GECCO) also assessed G×BMI interactions and discovered a new locus located within the *FMN1/GREM1* gene region (rs58349661) that interacts with BMI in the association with colorectal cancer risk^188^. Due to the substantial risk for false discovery and ensuing penalties for multiple comparisons, the discovery of new G×E interactions may be limited by statistical power and sample size requirements.

### 4.5. Differences by cancer site

In our meta-analysis there were large differences in the magnitudes of associations of BMI with risk of different cancers, suggesting diverse mechanistic underpinnings of adiposity relationships. The largest effect sizes were observed for oesophageal adenocarcinoma, which has been hypothesised to be due to the effect of excess weight compressing the stomach, which increases the risk of gastro-oesophageal reflux disease^189^. Other organs susceptible to metabolic disorders, such as kidney (hypertension), gallbladder (gallstones), liver and pancreatic cancers (non-alcoholic fatty liver disease, and diabetes) also had strong associations with BMI. Adiposity is an important determinant of oestradiol, which is a known risk factor for female reproductive cancers such as breast and endometrial cancer^90,94–97^. While for other cancers underlying associations may be less direct, such as mediated through inflammatory processes and other downstream effects of adiposity and therefore associations may plausibly be weaker.

### 4.6. Differences by tumour subtypes

Tumours can be classified according to histological, molecular, and mutation-defined subtypes, which can have prognostic value, but may also offer insights into aetiological pathways. Large-scale and comprehensive analyses of adiposity and cancer relationships stratified by tumour molecular pathological characteristics are now being explored to better understand the biological pathways underlying adiposity-related tumorigenesis. Such an approach has the potential to improve understanding of causal effects and link obesity to specific somatic molecular changes in tumours and provide refined risk estimates for newly classified cancer subtypes. For example, obesity is a stronger risk factor for ER+ breast tumours in postmenopausal women^181^. A handful of relatively small studies have found that the BMI and colorectal cancer relationship differed according to tumour molecular characteristics^190,191^. However, a recent study which analysed >11,800 cases found limited evidence of heterogeneity for the association between BMI and colorectal cancer risk according to major molecular subtypes, suggesting that obesity influences nearly all major pathways involved in colorectal carcinogenesis^192^. Obesity has been associated with elevated risk of KRAS-mutant, but not KRAS-wild-type colorectal tumours^192^.

## 5. Future directions

As our understanding of the relationships between excess adiposity and cancer continues to evolve, recent advances present novel opportunities to underlying mechanisms and to translate these insights into cancer prevention strategies.

### 5.1. Pharmacological opportunities

There is accelerating interest in pharmacological agents for weight loss, such as incretin mimetics. These drugs are a new class of promising non-surgical interventions, with randomised controlled trials reporting substantial weight loss (up to 24% reduction in body weight in 48 weeks)^193^. The impact of these pharmaceutical agents on obesity treatment, and subsequent effects on health and the biological pathways known to underlie the obesity-cancer relationship is likely to become a major research interest. A recent pragmatic randomised controlled trial, specifically the RECOVERY trial, has exemplified the potential for conducting large-scale trials efficiently by leveraging routinely collected healthcare data^194,195^. Considering the observed safety profile of these novel obesity treatments, the feasibility of a comprehensive randomised controlled trial assessing weight loss and its repercussions on health may be plausible. Alternatively, a natural experiment may be viable if access to these drugs remain inconsistent by healthcare/insurance providers.

### 5.2. Diverse populations

Our meta-analysis demonstrated almost negligible prospective data on BMI and cancer risk outside of North America, Europe, and East Asia. Expansion of global data, coupled with efforts to broaden the diversity of populations enrolled within studies, is increasingly recognised as a crucial step. Such expansion is essential to understand the complex interplay of genetic, environmental, and cultural factors that might influence obesity and health outcomes. Improved understanding will enhance healthcare forecasting, policy planning, and for formulating more effective prevention and intervention strategies that address specific challenges and contexts of these regions or populations. This approach is important to achieving more equitable global health improvements. To this aim, the development of robust population-based cancer and mortality registries, alongside improved healthcare infrastructure is essential to facilitate more comprehensive and accurate data collection and analysis^196–198^.

### 5.3. Imaging data

While data on BMI and waist circumference are widely available, computed tomography and magnetic resonance imaging technologies have only recently been deployed in large-scale prospective studies. The largest studies with imaging data available are the UK Biobank (target N=100,000) and the German National Cohort (N=30,000). The capability to directly measure fat mass, lean mass and fat depots provides a potentially more potent approach to delineate the precise relationship between adiposity and cancer. Notably, higher fat-free mass might decrease risk of some cancers (e.g. lung cancer)^26^. Therefore, fat-free mass could confound some BMI-cancer associations, potentially resulting in an underestimate of adiposity’s impact on risk of certain cancers. While data are still accruing in the UK Biobank, there is early evidence of the direct role of fat depots on site-specific cancer risk^199^. Imaging remains infeasible for many studies, but it may be possible to develop biological signatures of adiposity which might outperform BMI as an indicator of cancer risk and identify specific biomarkers of fat depots. If such signatures were demonstrated to be specific and generalisable, this could simplify research and extend study reach beyond imaging constraints.

### 5.4. Mendelian randomisation

The increasing volume of available genetic data provides greater breadth for MR approaches. To date, most studies have investigated genetically-predicted BMI as the primary instrument in obesity-cancer analyses. Some of the genetic variants in the BMI instrument may be associated with obesity via low lean mass or bone mineral density and may have heterogeneous associations with health^200^. Additionally, associations with adiposity distribution (e.g., subcutaneous adipose tissue and VAT) remain poorly characterised. In the future, it may be possible to cluster genetic variants into different groups based on their association with a wide variety of traits with greater precision, including high-throughput molecular markers, and thus tease apart the distinct genetic pathways leading to obesity and downstream health effects^201,202^. MR mediation analysis could also potentially be applied across thousands of biological factors. However, statistical power decreases with each additional mediator, which will be a major limitation. There are also opportunities stemming from the availability of whole genome sequencing, which will inform instrument selection and biological mechanisms for more targeted approaches.

### 5.5. Omics availability and casual inference

As the volume and complexity of epidemiological data continues to proliferate, the ability to process and interpret findings remains a challenge. The integration of multimodal data into a unifying coherent model could further provide insight into distinct components of obesity and their downstream effects on cancer risk, discover novel associations, and identify therapeutic candidates^203–205^. These developments present new challenges in harmonising disparate datasets, naming conventions and standardised frameworks to process the data. Furthermore, as our ability to identify tumour subtypes develops and the number of biomarkers investigated increases, ever larger sample sizes will be needed to penalise for multiple testing and detect effect modification^206^. Methods to address some of these complexities are being developed, including more advanced artificial intelligence and machine learning techniques. While machine learning processes efficiently process data and aid in the formulation of new hypotheses, limitations include bias, generalisability and a lack of transparency underlying model outputs^205,207,208^. Data collection in tumour subgroups and underrepresented populations is an important priority to avoid overfitting and reduce some biases.

Attributing causation also remains a challenge. The triangulation of data derived from orthogonal approaches to understanding the biological pathways linking obesity with cancer, for example human observational, genetic, and experimental models (pre-clinical, human organoids) has the potential to uncover novel mechanisms which could be targeted for precision prevention amongst those at higher risk of obesity-related cancer^34^.

### 5.6. Tissue samples

In addition to investigating the association of obesity with the molecular characteristics of malignant tumour tissue, conducting analyses on non-malignant tissues collected from individuals who are normal weight or who are overweight or obese could offer fresh insights into the biology underlying the initial stages of adiposity-related tumorigenesis. This approach could enable investigations of the abundance and upregulation of adiposity–cancer-related pathways at the target tissue level. Collection of normal tissues from patients undergoing weight reduction surgery, pharmacological interventions, or enrolled in diet-induced weight loss programs could also offer a resource for understanding mechanistic pathways at the target tissue level and ongoing studies have already provided novel insights into potential underlying pathways^209–211^.

### 5.7. Routinely collected data

Our meta-analysis demonstrated a growing prevalence of studies based entirely on routinely collected data, which have notably larger sample sizes than prospective cohort studies. While this approach maximises statistical power and generalisability, challenges persist in ensuring concurrent data collection of variables such as weight and smoking as well as important covariate data. However, there is ongoing expansion in the breadth of data collected and integration across different databases^212,213^. Routine data collection is also facilitating increasingly large cohorts, such as Our Future Health, which aims to recruit 5 million participants^214^. Such expansion may also eventually help to address some limitations in statistical power associated with multi-omics and cancer subtype analyses.

## 6. Clinical and policy implications

### 6.1. Public health policy

No country has succeeded in reversing their obesity trends. Current policies include promoting healthier food choices and physical activity and reducing unhealthy foods via education and taxes/subsidies^215^. Taxes on sugar-sweetened beverages are increasingly applied and have been shown to reduce the purchase of sugary drinks or incentivise reformulation, but the direct effects on obesity are difficult to measure^216^. Despite these efforts, adverse obesity trends persist, demonstrating that policies have been insufficient. Policy makers should continue to develop strategies which address the modifiable systemic factors that influence weight gain, such as obesogenic environments and the commercial determinants of health^217^. However, given the limited success of policies, radical new approaches to tackle obesity might also be necessary. Given the “stickiness” of obesity once onset, for individuals where prevention measures have not succeeded, greater acceptance of medical interventions may be required.

### 6.2. Obesity treatment

The emergence of incretin mimetics presents an unprecedented opportunity to treat obesity with improvement in health outcomes. However, long-term treatment may be required due to known weight regain following treatment withdrawal, and the high-cost barrier might inadvertently widen health disparities. Given the higher prevalence of obesity in low-income populations, there is need for careful considerations to ensure equitable health improvement. Furthermore, the modest treatment persistence rate at 1-year (61.8%, 95% CI 57.8–65.7%) underscores challenges in sustaining adherence^218^. Given these limitations in current pharmaceutical weight loss interventions, identifying potential therapeutic targets which might offset some of the harmful metabolic effects of obesity is likely to remain an important research priority.

### 6.3. Targeted screening

Due to the broad spectrum of diseases associated with obesity, any policy regarding targeted intervention needs to consider an individual’s risk associated with a variety of health outcomes, including but not limited to cancer risk. Towards this goal, future efforts are needed to develop models for predicting absolute risk of a broad range of health outcomes, such as risk of at least one of several obesity associated diseases, overall mortality, or disability adjusted life-years. Such models can inform identification of individuals who will benefit most from interventions based on, not only their current obesity status, but also taking into consideration other risk factors, such as age, gender, social determinants of health and lifestyle-related factors, which broadly contribute to health. Novel approaches are also being developed which integrate risk estimates derived from MR studies into observational risk prediction models. This integration will enhance the accuracy and reliability of forecasts regarding the effects of targeted obesity interventions, both for individuals and different populations categorised by risk group^219^.

## 7. Conclusion

We report that excess adiposity is positively associated with 19 common types of cancer. However, >99% of the worldwide prospective cohort data available are from North America, Europe, and East Asia, limiting our understanding of the obesity-cancer relationship in other populations. Recent transformative technological advances present novel opportunities to identify novel mechanistic mediators, investigate associations by tumour subtypes, and evaluate adiposity beyond standard anthropometric indicators. Such advances have the potential to significantly enhance our understanding of how obesity causes cancer and provide the evidence base for new preventative strategies and the formulation of effective public health policies.

## Supplementary Material

Supplement 1

Supplement 2

## Figures and Tables

**Figure 4A:**
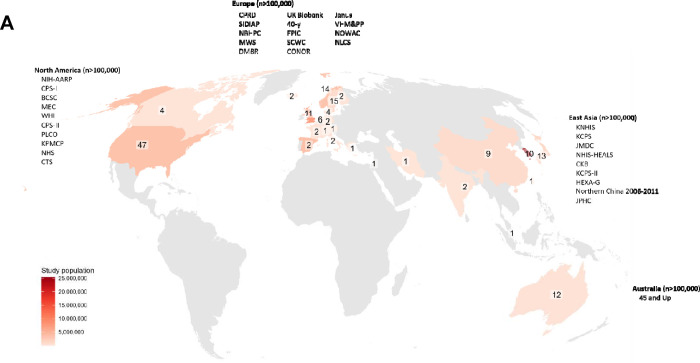
World map of included cohorts Numbers show the number of prospective cohorts with cancer data in each country included in this meta-analysis. Study population is the sum of cohort participants within each country. For cohorts with different analytic cohort sizes in different publications, the largest analytic size was used. Text labels list prospective studies with >100,000 participants for each region. Abbreviations: 40-y=40-year cohort, 45 and Up=The Sax Institute’s 45 and Up Study, BCSC=Breast Cancer Surveillance Consortium, CKB=China Kadoorie Biobank, CONOR=Cohort of Norway, CPRD=UK Clinical Practice Research Datalink, CPS=Cancer Prevention Study, CTS=California Teachers Study, DMBR=Danish Medical Birth Registry, EPIC=European Prospective Study into Cancer and Nutrition, HEXA-G=Health Examinees-Gem, JPHC=Japan Public Health Center, KCPS=Korean Cancer Prevention Study, KNHIS=National Health Insurance Service of Korea, KPMCP=Kaiser Permanente Medical Care Program, MEC=Multiethnic Cohort Study, MWS=Million Women Study, NBHPC=Norwegian BMI/Height Prospective Cohort 1963–2001, NHIS-HEALS=National Health Insurance Service-National Health Screening Cohort, NHS=Nurses’ Health Study, NIH-AARP=National Institutes of Health-AARP, NLCS=Netherlands Cohort Study, NOWAC=Norwegian Women and Cancer Study, PLCO=Prostate, Lung, Colorectal and Ovarian Cancer Screening Trial, SCWC=Swedish Construction Workers Cohort, SIDIAP=Information System for Research in Primary Care, WHI=Women’s Health Initiative, VHM&PP=Vorarlberg Health Monitoring and Prevention Programme

**Figure 4B:**
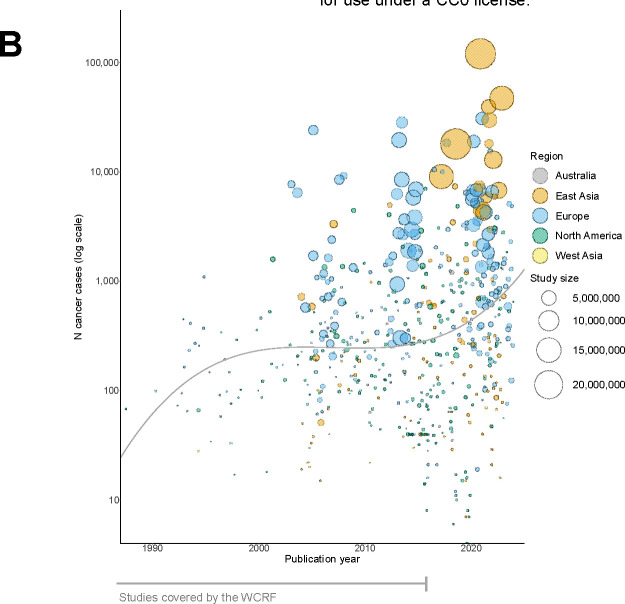
Prospective cancer studies by N incident cancer cases and publication year The size of each bubble is proportional to the size of the analytic cohort. Bubbles show the number of participants within each BMI-cancer study and represent the most recent data available for each BMI-cancer association (i.e., we excluded studies using duplicate cohorts with fewer site-specific cancers). Trend line represents the average number of cases per year, modelled by polynomial spline. The upward trend over time is driven primarily by the addition of large healthcare databases but also partly reflect our sample size criterion (n>50,000) for studies published after the WCRF reports. Abbreviations: WCRF=World Cancer Research Fund.

## Box 1: Mediation analysis

Statistical mediation analysis is a useful tool for identifying biological factors linking obesity and cancer risk. Unlike experimental analyses that involve direct manipulation of study factors in animal models, mediation analysis instead uses formal statistical models to identify mediators. While there are key assumptions inherent in mediation analysis, this model-based approach is highly scalable and can be derived from human data, helping to ensure results apply to populations.

Traditionally, mediation analysis compares exposure-disease effects from two regression models, one without adjustment for mediators and one with adjustment. As applied to obesity and cancer, the “total effect” is the obesity-cancer association without meditator adjustment while the “direct effect” is the association with such adjustment. The percent of excess risk that a mediator explains is the difference between these (total effect-direct effect) divided by the total effect. The percent excess risk integrates information on the strength of obesity-biomarker correlations and biomarker-cancer associations into a single measure that can then be used to rapidly rank biomarkers for mediation potential.

Mediation analysis assumes that there is no unmeasured confounding between the mediator, exposure and outcome, and no intermediate confounders^87^ —which may not hold given the 100 to 1000s of biological factors related to obesity. In this context, any given candidate mediator may correlate with dozens of other unmeasured cancer-related biological factors, thus giving rise to confounding. To better determine causal factors, mediation analyses should consider using high-throughput measures (e.g. metabolomics, proteomics) to permit a comprehensive adjustment of biological factors. MR based mediation analysis offers a complementary approach to observational methods and may reduce some concerns about confounding^88^.
