# Adiposity and cancer: systematic review and meta-analysis

**DOI:** 10.1101/2024.02.16.24302944

**Published:** 2025-09-16

**Authors:** Eleanor L. Watts, Amparo Gonzalez-Feliciano, Marc J. Gunter, Nilanjan Chatterjee, Steven C. Moore

## Abstract

Obesity is a growing global health challenge and a major cancer risk factor. We conducted a systematic review and meta-analysis of prospective cohorts quantifying associations between body mass index (BMI) and 25 common cancers, searching PubMed, EMBASE and Scopus through April 23rd, 2025. Across 226 articles comprising 1.5 million cancer data points, BMI was positively associated with 19 cancer types and inversely associated with three. We identified positive associations for leukaemia, non-Hodgkin lymphoma, bladder, and glioma—novel findings which were not reported by major consensus reports. Regional differences were observed, including stronger associations for postmenopausal breast and ovarian cancers in East Asia, and weaker associations for gallbladder cancer. BMI and waist circumference showed similar associations with cancer. We also reviewed Mendelian randomisation studies and emerging imaging-based evidence; genetic data largely supported causality, while imaging-data were limited. Our findings underscore the broad and growing impact of obesity on cancer risk.

## Full Text Availability

The license terms selected by the author(s) for this preprint version do not permit archiving in PMC. The full text is available from the preprint server.

