# Supplementary material for: Adiposity and cancer: meta-analysis, mechanisms, and future perspectives
Source: medRxiv. 2024 Feb 18:2024.02.16.24302944. Preprint. [Version 1] doi: 10.1101/2024.02.16.24302944 (PMC10889047; doi:10.1101/2024.02.16.24302944)
Supplement: Supplementary file 1 [file media-2.docx]

**Adiposity and cancer: meta-analysis, mechanisms, and future perspectives**

**Supplementary materials**

**Table of Contents**

Supplementary Methods	5

1.	Cancer selection	5

2.	Study selection and literature search	5

*i.*	*Studies included in the WCRF reviews*	2

*ii.*	*Studies published after the WCRF reviews*	2

3.	Verifying completeness	3

4.	Data extraction	3

5.	Risk harmonisation	4

6.	Meta-analysis	4

7.	Heterogeneity by region	5

8.	Heterogeneity in the associations of BMI and waist circumference with cancer risk	5

9.	Sensitivity analyses	5

10.	Mendelian randomisation studies	5

11.	References	89

**Supplementary Tables**

Supplementary Table 1: Publications identified and inclusion criteria by cancer type	6

Supplementary Table 2: PubMed search terms	7

Supplementary Table 3: Comparison of current meta-analysis results with WCRF reviews	11

Supplementary Table 4: Pooled estimates with and without large health databases included	12

**Supplementary Figures** 

	[Supplementary Figure 1: Studies identified by search strategy*13](https://nih-my.sharepoint.com/personal/wattsel_nih_gov/Documents/Reviews/Obesity%20and%20cancer%20risk/Submission/Rev1/Supplementary_materials_11082023.docx)

	[Supplementary Figure 2: Percentage of cancer cases originating from each geographic region14](https://nih-my.sharepoint.com/personal/wattsel_nih_gov/Documents/Reviews/Obesity%20and%20cancer%20risk/Submission/Rev1/Supplementary_materials_11082023.docx)

Supplementary Figure 3: Meta-analysis of prospective studies on the risk of endometrial cancer in relation to BMI	15

Supplementary Figure 4: Meta-analysis of prospective studies on the risk of oesophageal adenocarcinoma cancer in relation to BMI	16

Supplementary Figure 5: Meta-analysis of prospective studies on the risk of kidney cancer in relation to BMI	17

Supplementary Figure 6: Meta-analysis of prospective studies on the risk of liver cancer in relation to BMI	18

Supplementary Figure 7: Meta-analysis of prospective studies on the risk of gastric cancer (cardia) in relation to BMI	19

Supplementary Figure 8: Meta-analysis of prospective studies on the risk of gallbladder cancer in relation to BMI	20

Supplementary Figure 9: Meta-analysis of prospective studies on the risk of meningioma in relation to BMI	21

Supplementary Figure 10: Meta-analysis of prospective studies on the risk of breast (postmenopausal) cancer in relation to BMI	22

Supplementary Figure 11: Meta-analysis of prospective studies on the risk of head and neck cancer (restricted to never-smokers) in relation to BMI	23

Supplementary Figure 12: Meta-analysis of prospective studies on the risk of thyroid cancer in relation to BMI	24

Figure 13: Meta-analysis of prospective studies on the risk of colorectal cancer in relation to BMI	25

Supplementary Figure 14: Meta-analysis of prospective studies on the risk of pancreatic cancer in relation to BMI	26

Supplementary Figure 15: Meta-analysis of prospective studies on the risk of leukaemia in relation to BMI	27

Supplementary Figure 16: Meta-analysis of prospective studies on the risk of multiple myeloma in relation to BMI	28

Supplementary Figure 17: Meta-analysis of prospective studies on the risk of ovarian cancer in relation to BMI	29

Supplementary Figure 18: Meta-analysis of prospective studies on the risk of prostate cancer (aggressive) in relation to BMI	30

Supplementary Figure 19: Meta-analysis of prospective studies on the risk of bladder cancer (restricted to never-smokers) in relation to BMI	31

Supplementary Figure 20: Meta-analysis of prospective studies on the risk of NHL in relation to BMI	32

Supplementary Figure 21: Meta-analysis of prospective studies on the risk of cervical cancer in relation to BMI	33

Supplementary Figure 22: Meta-analysis of prospective studies on the risk of glioma in relation to BMI	34

Supplementary Figure 23: Meta-analysis of prospective studies on the risk of melanoma in relation to BMI	35

Supplementary Figure 24: Meta-analysis of prospective studies on the risk of lung cancer (restricted to never-smokers) in relation to BMI	36

Supplementary Figure 25: Meta-analysis of prospective studies on the risk of gastric cancer (non-cardia) in relation to BMI	37

Supplementary Figure 26: Meta-analysis of prospective studies on the risk of breast cancer (premenopausal) in relation to BMI	38

Supplementary Figure 27: Meta-analysis of prospective studies on the risk of oesophageal (SQ, restricted to never-smokers) in relation to BMI	39

Supplementary Figure 28: Meta-analysis of prospective studies on the risk of endometrial cancer in relation to BMI, by region	40

Supplementary Figure 29: Meta-analysis of prospective studies on the risk of oesophageal adenocarcinoma cancer in relation to BMI, by region	41

Supplementary Figure 30: Meta-analysis of prospective studies on the risk of kidney cancer in relation to BMI, by region	42

Supplementary Figure 31: Meta-analysis of prospective studies on the risk of liver cancer in relation to BMI, by region	43

Supplementary Figure 32: Meta-analysis of prospective studies on the risk of gastric cancer (cardia) in relation to BMI, by region	44

Supplementary Figure 33: Meta-analysis of prospective studies on the risk of gallbladder cancer in relation to BMI, by region	45

Supplementary Figure 34: Meta-analysis of prospective studies on the risk of meningioma cancer in relation to BMI, by region	46

Figure 35: Meta-analysis of prospective studies on the risk of breast cancer (postmenopausal) in relation to BMI, by region	47

Supplementary Figure 36: Meta-analysis of prospective studies on the risk of head and neck cancer (restricted to never-smokers) in relation to BMI, by region	48

Supplementary Figure 37: Meta-analysis of prospective studies on the risk of thyroid cancer in relation to BMI, by region	49

Supplementary Figure 38: Meta-analysis of prospective studies on the risk of colorectal cancer in relation to BMI, by region	50

Supplementary Figure 39: Meta-analysis of prospective studies on the risk of pancreatic cancer in relation to BMI, by region	51

Supplementary Figure 40: Meta-analysis of prospective studies on the risk of leukaemia in relation to BMI, by region	52

Supplementary Figure 41: Meta-analysis of prospective studies on the risk of multiple myeloma in relation to BMI, by region	53

Supplementary Figure 42: Meta-analysis of prospective studies on the risk of ovarian cancer in relation to BMI, by region	54

Supplementary Figure 43: Meta-analysis of prospective studies on the risk of aggressive prostate cancer in relation to BMI, by region	55

Supplementary Figure 44: Meta-analysis of prospective studies on the risk of bladder cancer (restricted to never-smokers) in relation to BMI, by region	56

Supplementary Figure 45: Meta-analysis of prospective studies on the risk of NHL in relation to BMI, by region	57

Supplementary Figure 46: Meta-analysis of prospective studies on the risk of cervical cancer in relation to BMI, by region	58

Supplementary Figure 47: Meta-analysis of prospective studies on the risk of glioma in relation to BMI, by region	59

Supplementary Figure 48: Meta-analysis of prospective studies on the risk of melanoma in relation to BMI, by region	60

Supplementary Figure 49: Meta-analysis of prospective studies on the risk of lung cancer (restricted to never-smokers) in relation to BMI, by region	61

Supplementary Figure 50: Meta-analysis of prospective studies on the risk of gastric cancer (non-cardia) in relation to BMI, by region	62

Supplementary Figure 51: Meta-analysis of prospective studies on the risk of breast cancer (premenopausal) in relation to BMI, by region	63

Supplementary Figure 52: Meta-analysis of prospective studies on the risk of oesophageal SQ in relation to BMI, by region	64

Supplementary Figure 53: Associations of BMI, waist circumference and endometrial cancer risk, per 1 SD increase	65

Supplementary Figure 54: Associations of BMI, waist circumference and oesophageal adenocarcinoma risk, per 1 SD increase	66

Supplementary Figure 55: Associations of BMI, waist circumference and liver cancer risk, per 1 SD increase	67

Supplementary Figure 56: Associations of BMI, waist circumference and kidney cancer risk, per 1 SD increase	68

Supplementary Figure 57: Associations of BMI, waist circumference and gallbladder cancer risk, per 1 SD increase	69

Supplementary Figure 58: Associations of BMI, waist circumference and gastric cancer (cardia) risk, per 1 SD increase	70

Supplementary Figure 59: Associations of BMI, waist circumference and breast (postmenopausal) cancer risk, per 1 SD increase	71

Supplementary Figure 60: Associations of BMI, waist circumference and pancreatic cancer risk, per 1 SD increase	72

Supplementary Figure 61: Associations of BMI, waist circumference and head and neck cancer risk, per 1 SD increase	73

Supplementary Figure 62: Associations of BMI, waist circumference and thyroid cancer risk, per 1 SD increase	74

Supplementary Figure 63: Associations of BMI, waist circumference and colorectal cancer risk, per 1 SD increase	75

Supplementary Figure 64: Associations of BMI, waist circumference and leukaemia risk, per 1 SD increase	76

Supplementary Figure 65: Associations of BMI, waist circumference and multiple myeloma risk, per 1 SD increase	77

Supplementary Figure 66: Associations of BMI, waist circumference and ovarian cancer risk, per 1 SD increase	78

Supplementary Figure 67: Associations of BMI, waist circumference and NHL risk, per 1 SD increase	79

Supplementary Figure 68: Associations of BMI, waist circumference and melanoma risk, per 1 SD increase	80

Supplementary Figure 69: Associations of BMI, waist circumference and bladder cancer risk, per 1 SD increase	81

Supplementary Figure 70: Associations of BMI, waist circumference and prostate cancer risk, per 1 SD increase	82

Supplementary Figure 71: Associations of BMI, waist circumference and cervical cancer risk, per 1 SD increase	83

Supplementary Figure 72: Associations of BMI, waist circumference and gastric cancer (non-cardia) risk, per 1 SD increase	84

Supplementary Figure 73: Associations of BMI, waist circumference and lung cancer risk, per 1 SD increase	85

Supplementary Figure 74: Associations of BMI, waist circumference and lung cancer risk (restricted to never-smokers), per 1 SD increase	86

Supplementary Figure 75: Associations of BMI, waist circumference and breast (premenopausal) cancer risk, per 1 SD increase	87

Supplementary Figure 76: Associations of BMI, waist circumference and oesophageal SQ cancer risk, per 1 SD increase	88

# **Supplementary Methods**

## 1. **Cancer selection**

We selected cancer types *a priori* based on the 25 most common cancers by global incidence (https://www.wcrf.org/cancer-trends/worldwide-cancer-data/). Among these, we grouped cancers of the lip, oral cavity, larynx, nasopharynx, oropharynx, and hypopharynx as head and neck cancers per common convention. We subdivided cancers known to have etiologically distinct subtypes (“subtype-specific cancers”) as follows: gastric (cardia, non-cardia), breast (pre- and post-menopausal), oesophageal (adenocarcinoma and squamous cell carcinoma), and central nervous system (CNS; glioma and meningioma). 

For two groups of cancers—smoking-related cancers and prostate cancer—we conducted special analyses designed to address biases of particular importance for these cancers. For the cancers most strongly related to smoking (head and neck, lung, oesophageal squamous cell carcinoma (SQ) and bladder), per Freedman et al.^1^, we restricted analyses to never smokers to eliminate or reduce residual confounding by smoking. For prostate cancer, we restricted analyses to aggressive cancers to minimize screening bias, which can occur when healthier men are more likely to be screened and have indolent prostate cancers detected^2^. Aggressive prostate cancer was defined as any of stage 3-4 on the American Joint Committee on Cancer 1992 classification, advanced cancer, advanced or metastatic cancer; metastatic cancer; stage C or D on the Whitmore/Jewett scale; fatal cancer (prostate cancer-specific mortality); high stage or grade; Gleason grade ≥ 7). This definition was based on previous WCRF reviews^3^. 

Ultimately, the following 25 cancer types were investigated: bladder (never-smokers only), breast cancer (pre and postmenopausal), cervix, CNS (glioma and meningioma), colorectal, endometrial, gallbladder, gastric (non-cardia and cardia), head and neck (never-smokers only), kidney, leukaemia, liver, lung (never smokers only), melanoma, multiple myeloma, non-Hodgkin lymphoma (NHL), oesophageal SQ (never-smokers only), oesophageal adenocarcinoma, ovarian, pancreas, prostate (aggressive), and thyroid.

## 2. **Study selection and literature search**

Studies were identified using two separate search paths: i) studies included in the World Cancer Research Fund (WCRF) expert reviews (or a recent meta-analysis if not available), and ii) studies published after the last included study within each review. 

### i. *Studies included in the WCRF reviews*

For each cancer site, we identified studies of BMI and cancer risk which were included in the WCRF reviews^3–18^.  For cancer sites not included by the WCRF (leukaemia, multiple myeloma, NHL, thyroid, meningioma, and glioma) we followed the same protocol using recent relevant meta-analyses for these sites^19–21^.  

#### WCRF study exclusions

In total the WCRF reviews and selected meta-analysis included 492 studies of BMI and cancer risk, the number of studies for each site ranged from 8 (gallbladder and liver cancers) to 42 (postmenopausal breast cancer) (**Supplementary Table 1**).

For studies identified by the WCRF reviews or meta-analyses, studies were excluded based on the following criteria: 

1) Non-cancer incidence outcome (e.g., mortality) or non-prospective design.

2) Risk estimates were available from more recent publications with a greater number of cases.

3) No risk estimates for never-smokers for smoking-related cancers (for bladder cancer associations restricted to never-smokers were not reported in the WCRF review).

### ii. *Studies published after the WCRF reviews*

The last study included in each WCRF review varied by site, ranging from 2008 (gastric cancer) to 2016 (melanoma) (**Supplementary Table 3**). Studies published since each of the selected reviews were identified using a PubMed search, starting at the year of the last included study for each cancer site. PubMed search terms are available from **Supplementary Table 2**. 

#### Exclusions for studies published after the WCRF reviews

In total our PubMed search identified 1,632 publications (**Supplementary Figure 1**), the number site-specific publications ranged from 6 (NHL) to 247 (breast cancer) (**Supplementary Table 1**). 

Studies were excluded based on the following criteria:

1) Non-cancer incidence outcome (e.g., survival/prognosis/wrong outcome) or non-prospective study design.

2) N <50,000 participants in the study cohort (unless an updated estimate from a previously included cohort included in initial WCRF/meta-analysis or included in an identified pooled analysis with total participants >50,000). 

3) Risk estimates not convertible to continuous estimates, such as where only two BMI categories were reported (e.g., risk estimates comparing obese and non-obese participants only).

4) No risk estimates available for subtypes for subtype-specific cancers. In East and Southeast Asia >90% of oesophageal cancers are SQ and >80% of gastric cancers are non-cardia^22^, therefore for cohorts in this region, cancers were categorised into these respective subtypes if not otherwise stated.

5) No risk estimates for never-smokers for smoking-related cancers (head and neck, lung, oesophageal SQ and bladder).

6) No adjustment for smoking or evaluation of confounding by smoking.

7) Risk estimates were based on models which adjusted for other measures of adiposity (such as waist circumference) or early-life body size only (due to collinearity of variables with adult BMI).

8) Duplicate study population. Where one cohort had multiple publications identified, the publication with the greatest number of cases was included. 

9) Duplicate publication. Overlap between the study identified by Pubmed search and the WCRF/meta-analysis (this occurred because our search dates include the year that the last study in the WCRF was published). 

Where studies were identified in the literature search which reported results for multiple cancers, BMI cancer associations were extracted for each reported site and screened using the process outlined above (N=31 publications). The number of studies excluded for each exclusion criteria and cancer type are available from **Supplementary Table 1.**

## 3. **Verifying completeness**

To ensure our literature search was complete, we searched reference lists of studies published post-WCRF. Specifically, we searched the reference lists of Larrson et al.^23^, which included a narrative review of studies published from 2018-2021, and the most recent papers for each cancer type in our literature search. Reference lists were searched for study titles that noted a measure of adiposity and a cancer of interest. We then excluded studies based on the criteria outlined above. In total, we identified three additional publications from reference review (for endometrial and colorectal cancers)^24–26^. 

## 4. **Data extraction**

For each study, we extracted risk estimates (hazard ratios (HRs), risk ratios (RRs) or odds ratios (ORs) and their 95% confidence intervals (CIs) for the association of BMI with incident cancer risk, preferentially selecting estimates based on a continuous modelling of BMI. If continuous risk estimates were unavailable and BMI had at least three categories, we extracted categorical estimates. We extracted the estimates with the most comprehensive adjustments for confounders that did not also adjust for other measures of adiposity (such as waist circumference), BMI from an earlier age, or height (as height is part of the equation for estimating BMI which could complicate interpretation of height-adjusted models).

Where estimates originated from pooled analyses, we extracted study-specific risk estimates, if possible, unless cohorts were judged to be sufficiently similar in design (e.g., NHS, NHS2 and HPFS or EPIC countries). Where only pooled estimates were available in each article, we contacted the corresponding authors for study-specific estimates. If study-specific estimates remained unavailable, pooled estimates were included if the number of cases in the pooled study exceeded the individual study publications. These were made available to us in some instances^27–29^. If multiple studies were available from analyses of national health databases (e.g., Korean National Health Insurance Service (KNHIS) database), risk estimates from the analysis with the largest number of cases was included to reduce the possibility of including the same participants multiple times. Where studies included individual risk estimates for cancer subsites and not the overall associations, risk estimates for each site were included based on conventions within the field and if there was no overlap between cases (e.g., where a study reported risk estimates for colon and rectal cancer separately both risk estimates for both sites were extracted)^14^. 

## 5. **Risk harmonisation**

Risk estimates were converted to the 5 kg/m^2 ^scale to enable comparison across studies. Where estimates were reported in a different continuous scale (e.g., per 1 standard deviation (SD)), HRs and 95% CIs were rescaled to HR per 5 kg/m^2 ^using the formula: HR per 5 kg/m^2^= 𝐻𝑅_𝑥_^5/𝑥^, where 𝑥 is equal to the originally reported units. The same method was used for the upper and lower CIs. Where HRs were reported as categorical estimates, associations were converted to the continuous scale using methods described in detail elsewhere^30^. In brief, midpoints of each BMI group were calculated unless directly reported in the article with upper and lower bounds assigned based on the study population. Trend was estimated using generalised least squares using the ‘dosresmeta’ R package (R version 4.3.1)^31^. 

## 6. **Meta-analysis**

Summary risk estimates for the association of BMI with each cancer site were estimated using random effects meta-analysis, which weights studies based on within-study and between-study variability and calculated a weighted average of individual study effect size. These estimates were calculated using the ‘meta’ R package^32^. Heterogeneity across studies was assessed using I^2^, tau^2^, and the 25th and 75th percentile (interquartile range) of RRs for each cancer site. I^2^ estimates the percent of total variation due to true differences in effect sizes between studies; an I^2 ^close to 100% indicates that chance plays little role in observed differences. However, I^2^ is highly sensitive to sample size— when studies are large, even small differences can yield large I^2 ^values ^33^. Tau^2^ more directly quantifies differences in effect sizes—it is the between-study variance of the estimated underlying distribution of true effect sizes. A value close to 0 suggests that the true effect size varies little between populations. This metric was calculated using the Paule-Mandel estimator^34^. Finally, we present the interquartile range as a more intuitive metric, showing the typical spread of estimates.

## 7. **Heterogeneity by region**

We assessed heterogeneity in the associations of BMI and each cancer site by region. Cohorts were clustered into geographic regions: Australia (Australia), North America (USA and Canada), Europe (Austria, Denmark, Finland, France, Germany, Greece, Iceland, Italy, Netherlands, Norway, Spain, Sweden, Switzerland, UK), East Asia (Japan, South Korea, China, Taiwan, Singapore), South Asia (India), West Asia (Iran and Israel)). Although Singapore is geographically located in Southeast Asia, the only study identified was the Singapore Chinese Health Study, which had a relatively small study population (N=51,000) and was specifically recruited from the Chinese population of Singapore. Therefore, Singapore was grouped with East Asia. Heterogeneity in the associations by geographic region was quantified using the Q-statistic.

## 8. **Heterogeneity in the associations of BMI and waist circumference with cancer risk**

The associations of BMI and waist circumference were based on the same population; therefore, heterogeneity was tested using the Wald statistic:

$Wald statistic= \frac{β_{1}- β_{2}}{\sqrt{Var(β_{1})- Var(β_{2})-2Cov(β_{1},β_{2})}}$

Where: 

$β_{1}$ = log RR per 1 standard deviation increase in BMI

$β_{2}$= log RR per 1 standard deviation increase in waist circumference

$Cov$ = *r* $×\sqrt{Var(β_{1}) × Var(β_{2}) }$ 

*r* = correlation coefficient of BMI and waist circumference (*r* = 0.87)^35^ 

## 9. **Sensitivity analyses**

Some of the individual study estimates were calculated entirely from national health databases, rather than prospective cohorts, which generally have more limited ability to control for residual confounding (e.g., Clinical Practice Research Datalink (CPRD, 50% missing smoking information))^36^ and often are not linked to gold-standard cancer registry data, increasing misclassification biases^37^. These databases often comprise very large populations (e.g., KNHIS, up to 23 million population)^38^ and so could influence pooled estimates. Therefore, in sensitivity analyses these health databases were excluded (KNHIS, CPRD, and Information System for Research in Primary Care)^36,38,39^.

## 10. **Mendelian randomisation studies**

For each of the sites included in this meta-analysis, we also identified Mendelian randomisation (MR) studies with the largest number of cases currently available and drawn from single genetic ancestry populations to reduce confounding by population structure.

Supplementary Tables

Supplementary Table 1: Publications identified and inclusion criteria by cancer type

| Cancer site | Study identification |  |  | Exclusions for studies after WCRF |  |  |  |  |  |  |  |  | Total included studies |
| --- | --- | --- | --- | --- | --- | --- | --- | --- | --- | --- | --- | --- | --- |
|  | WCRF/ meta-analysis cohorts | PubMed search | Other* | Non-cancer incidence outcome^a^ | N <50,000 in study cohort | Not convertible to cont estimate | No risk estimates available for subtypes† | No risk estimates for NS* | Did not evaluate confounding by smoking | HRs adj for adiposity measures | Duplicate cohort with fewer cases | Duplicate study |  |
| Melanoma | 14 | 14 | 7 | 11 | 0 | 0 | 0 | 0 | 0 | 0 | 4 | 2 | 18 |
| Thyroid | 22 | 29 | 7 | 16 | 0 | 1 | 0 | 0 | 0 | 0 | 11 | 1 | 29 |
| Breast (premenopausal) | 27 | 247 | 19 | 190 | 15 | 3 | 7 | 0 | 5 | 1 | 40 | 5 | 27 |
| Breast (postmenopausal) | 42 | 247 | 19 | 190 | 15 | 3 | 4 | 0 | 5 | 1 | 49 | 5 | 36 |
| Head and neck (NS) | 20 | 21 | 7 | 16 | 0 | 0 | 0 | 3 | 0 | 0 | 2 | 2 | 25 |
| Cervix | 9 | 15 | 8 | 14 | 0 | 0 | 0 | 0 | 1 | 0 | 2 | 3 | 12 |
| Leukaemia | 11 | 31 | 6 | 24 | 0 | 0 | 0 | 0 | 2 | 0 | 2 | 4 | 16 |
| Multiple myeloma | 16 | 19 | 6 | 12 | 2 | 0 | 0 | 0 | 2 | 0 | 6 | 3 | 16 |
| NHL | 19 | 6 | 4 | 6 | 0 | 0 | 0 | 0 | 0 | 0 | 1 | 1 | 21 |
| Colorectal | 29 | 179 | 18 | 123 | 15 | 5 | 0 | 0 | 3 | 0 | 34 | 3 | 43 |
| Glioma | 7 | 29 | 8 | 21 | 0 | 0 | 7 | 0 | 3 | 0 | 3 | 1 | 9 |
| Meningioma | 11 | 29 | 8 | 24 | 0 | 0 | 10 | 0 | 3 | 0 | 3 | 1 | 7 |
| Gallbladder | 8 | 19 | 8 | 13 | 1 | 0 | 0 | 0 | 0 | 0 | 4 | 4 | 13 |
| Liver | 8 | 61 | 13 | 40 | 1 | 3 | 0 | 0 | 3 | 1 | 15 | 2 | 17 |
| Ovary | 22 | 49 | 14 | 35 | 2 | 2 | 0 | 0 | 0 | 0 | 14 | 3 | 29 |
| Prostate (aggressive) | 23 | 116 | 10 | 83 | 13 | 0 | 12 | 0 | 1 | 1 | 12 | 1 | 26 |
| Bladder (NS) | 14 | 23 | 10 | 16 | 0 | 0 | 0 | 18 | 0 | 0 | 2 | 2 | 9 |
| Endometrial | 25 | 80 | 16 | 55 | 2 | 2 | 0 | 0 | 1 | 2 | 27 | 4 | 28 |
| Kidney | 23 | 44 | 14 | 36 | 1 | 0 | 0 | 0 | 2 | 0 | 15 | 2 | 25 |
| Pancreas | 23 | 53 | 16 | 31 | 3 | 0 | 0 | 0 | 4 | 0 | 25 | 1 | 28 |
| Gastric (cardia) | 7 | 46 | 10 | 23 | 2 | 1 | 9 | 0 | 2 | 0 | 11 | 2 | 13 |
| Gastric (non-cardia) | 7 | 46 | 10 | 23 | 2 | 1 | 6 | 0 | 2 | 0 | 11 | 2 | 16 |
| Oesophageal (adeno) | 9 | 89 | 11 | 70 | 2 | 1 | 5 | 4 | 2 | 0 | 10 | 3 | 12 |
| Oesophageal (SQ, NS) | 2 | 89 | 11 | 70 | 2 | 1 | 7 | 4 | 2 | 0 | 9 | 3 | 4 |
| Exclusions are sequential and so numbers sum across columns. Studies numbers reflect distinct PMIDs for each site, WCRF numbers reflect the number of cohorts. WCRF cohorts were excluded if they were not prospective or mortality was the outcome (exclusion criteria #1, except for aggressive prostate cancer where the mortality outcome criteria was not applied) or a larger duplicate cohort was available with more cases (#8). All other exclusion criteria were applied to the studies identified after WCRF reviews. *Identified from literature searches from the other cancers or reference review. †Oesophageal (adenocarcinoma, SQ), breast (pre and postmenopausal), prostate (aggressive), CNS (glioma and meningioma), gastric (cardia and non-cardia) only ‡Head and neck, lung, oesophageal (SQ) and bladder only. Abbreviations: CNS=central nervous system, HR=hazard ratio, NHL=non-Hodgkin lymphoma, NS=never-smokers, PMID=PubMed identifier, SQ=squamous cell carcinoma, WCRF=World Cancer Research Fund Lung (NS) | 12 | 51 | 11 | 41 | 3 | 0 | 0 | 7 | 0 | 0 | 7 | 3 | 13 |

Supplementary Table 2: PubMed search terms

| **Site** | **PubMed search terms** |
| --- | --- |
| Melanoma | ("melanoma"[MeSH] OR "melanoma*") AND ("body mass index"[ti] OR bmi[ti] OR ((obesity[ti] OR adipos*[ti] OR body fat*[ti] OR body weight[ti] OR body mass[ti] OR body composition[ti] OR lifestyle[ti] OR anthropom*[ti]) AND (bmi[tiab] OR "body mass index"[tiab:~0]))) AND (pool*[tiab] OR million[tiab] OR cohort*[tiab] OR risk*[ti] OR incidence[ti] OR associat*[ti] ) AND english[la] NOT (review[pt] OR "Case Reports"[pt] OR "Comment"[pt] OR "Health Education"[MeSH] OR "prediction model"[ti] OR surviv*[ti] OR patient*[ti] OR odds[tiab] OR "ORs"[tiab] ) NOT (Mendelian random*[ti] OR polymorphisms[ti] OR pathway*[ti] OR expression[ti] OR DNA[ti] OR microRNA[ti] OR mRNA[ti] OR polymorphism[ti] OR virus[ti]) AND 2016:2023[pdat] |
| [Thyroid](file:///C:\Users\wattsel\AppData\Local\Microsoft\Windows\INetCache\Content.MSO\CEED827C.xlsx) | ("thyroid neoplasms"[MeSH] OR "thyroid cancer") AND ("body mass index"[ti] OR bmi[ti] OR ((obesity[ti] OR adipos*[ti] OR body fat*[ti] OR body weight[ti] OR body mass[ti] OR body composition[ti] OR lifestyle[ti] OR anthropom*[ti]) AND (bmi[tiab] OR "body mass index"[tiab:~0]))) AND (pool*[tiab] OR million[tiab] OR cohort*[tiab] OR risk*[ti] OR incidence[ti] OR associat*[ti] ) AND english[la] NOT (review[pt] OR "Case Reports"[pt] OR "Comment"[pt] OR "Health Education"[MeSH] OR "prediction model"[ti] OR surviv*[ti] OR patient*[ti] OR odds[tiab] OR "ORs"[tiab] ) NOT (Mendelian random*[ti] OR polymorphisms[ti] OR pathway*[ti] OR expression[ti] OR DNA[ti] OR microRNA[ti] OR mRNA[ti] OR polymorphism[ti] OR virus[ti]) AND 2016:2023[pdat] |
| Breast | (breast neoplasms[MeSH] OR "breast cancer") AND ("body mass index"[ti] OR bmi[ti] OR ((obesity[ti] OR adipos*[ti] OR body fat*[ti] OR body weight[ti] OR body mass[ti] OR body composition[ti] OR lifestyle[ti] OR anthropom*[ti]) AND (bmi[tiab] OR "body mass index"[tiab:~0]))) AND (pool*[tiab] OR million[tiab] OR cohort*[tiab] OR risk*[ti] OR incidence[ti] OR associat*[ti] ) AND english[la] NOT (review[pt] OR "Case Reports"[pt] OR "Comment"[pt] OR "Health Education"[MeSH] OR "prediction model"[ti] OR surviv*[ti] OR patient*[ti] OR odds[tiab] OR "ORs"[tiab] ) NOT (Mendelian random*[ti] OR polymorphisms[ti] OR pathway*[ti] OR expression[ti] OR DNA[ti] OR microRNA[ti] OR mRNA[ti] OR polymorphism[ti] OR virus[ti]) AND 2015:2023[pdat] |
| Head and neck, lip and oral cavity | (nasopharyngeal neoplasms [MeSH] OR laryngeal neoplasms[MeSH] OR pharyngeal neoplasms[MeSH] OR mouth neoplasms[MeSH] OR tongue neoplasms[MeSH] OR oropharyngeal neoplasms[MeSH] OR hypopharyngeal neoplasms[MeSH] OR "head and neck cancer" OR "oral cancer" OR "larynx" OR "nasopharynx" OR "oropharynx" OR "hypopharynx" OR "pharynx") AND ("body mass index"[ti] OR bmi[ti] OR ((obesity[ti] OR adipos*[ti] OR body fat*[ti] OR body weight[ti] OR body mass[ti] OR body composition[ti] OR lifestyle[ti] OR anthropom*[ti]) AND (bmi[tiab] OR "body mass index"[tiab:~0]))) AND (pool*[tiab] OR million[tiab] OR cohort*[tiab] OR risk*[ti] OR incidence[ti] OR associat*[ti] ) AND english[la] NOT (review[pt] OR "Case Reports"[pt] OR "Comment"[pt] OR "Health Education"[MeSH] OR "prediction model"[ti] OR surviv*[ti] OR patient*[ti] OR odds[tiab] OR "ORs"[tiab] ) NOT (Mendelian random*[ti] OR polymorphisms[ti] OR pathway*[ti] OR expression[ti] OR DNA[ti] OR microRNA[ti] OR mRNA[ti] OR polymorphism[ti] OR virus[ti]) AND 2015:2023[pdat] |
| Cervix | ("uterine cervical neoplasms"[MeSH Terms] OR "cervical cancer") AND ("body mass index"[ti] OR bmi[ti] OR ((obesity[ti] OR adipos*[ti] OR body fat*[ti] OR body weight[ti] OR body mass[ti] OR body composition[ti] OR lifestyle[ti] OR anthropom*[ti]) AND (bmi[tiab] OR "body mass index"[tiab:~0]))) AND (pool*[tiab] OR million[tiab] OR cohort*[tiab] OR risk*[ti] OR incidence[ti] OR associat*[ti] ) AND english[la] NOT (review[pt] OR "Case Reports"[pt] OR "Comment"[pt] OR "Health Education"[MeSH] OR "prediction model"[ti] OR surviv*[ti] OR patient*[ti] OR odds[tiab] OR "ORs"[tiab] ) NOT (Mendelian random*[ti] OR polymorphisms[ti] OR pathway*[ti] OR expression[ti] OR DNA[ti] OR microRNA[ti] OR mRNA[ti] OR polymorphism[ti] OR virus[ti]) AND 2014:2023[pdat] |
| Leukaemia | ("leukemia"[MeSH] OR "leukemia*" OR "leukaemia*") AND ("body mass index"[ti] OR bmi[ti] OR ((obesity[ti] OR adipos*[ti] OR body fat*[ti] OR body weight[ti] OR body mass[ti] OR body composition[ti] OR lifestyle[ti] OR anthropom*[ti]) AND (bmi[tiab] OR "body mass index"[tiab:~0]))) AND (pool*[tiab] OR million[tiab] OR cohort*[tiab] OR risk*[ti] OR incidence[ti] OR associat*[ti] ) AND english[la] NOT (review[pt] OR "Case Reports"[pt] OR "Comment"[pt] OR "Health Education"[MeSH] OR "prediction model"[ti] OR surviv*[ti] OR patient*[ti] OR odds[tiab] OR "ORs"[tiab] ) NOT (Mendelian random*[ti] OR polymorphisms[ti] OR pathway*[ti] OR expression[ti] OR DNA[ti] OR microRNA[ti] OR mRNA[ti] OR polymorphism[ti] OR virus[ti]) AND 2014:2023[pdat] |
| Multiple myeloma | ("multiple myeloma"[MeSH] OR "multiple myeloma") AND ("body mass index"[ti] OR bmi[ti] OR ((obesity[ti] OR adipos*[ti] OR body fat*[ti] OR body weight[ti] OR body mass[ti] OR body composition[ti] OR lifestyle[ti] OR anthropom*[ti]) AND (bmi[tiab] OR "body mass index"[tiab:~0]))) AND (pool*[tiab] OR million[tiab] OR cohort*[tiab] OR risk*[ti] OR incidence[ti] OR associat*[ti] ) AND english[la] NOT (review[pt] OR "Case Reports"[pt] OR "Comment"[pt] OR "Health Education"[MeSH] OR "prediction model"[ti] OR surviv*[ti] OR patient*[ti] OR odds[tiab] OR "ORs"[tiab] ) NOT (Mendelian random*[ti] OR polymorphisms[ti] OR pathway*[ti] OR expression[ti] OR DNA[ti] OR microRNA[ti] OR mRNA[ti] OR polymorphism[ti] OR virus[ti]) AND 2014:2023[pdat] |
| Non-Hodgkin lymphoma | ("lymphoma, non hodgkin"[MeSH] OR "Non-Hodgkin lymphoma") AND ("body mass index"[ti] OR bmi[ti] OR ((obesity[ti] OR adipos*[ti] OR body fat*[ti] OR body weight[ti] OR body mass[ti] OR body composition[ti] OR lifestyle[ti] OR anthropom*[ti]) AND (bmi[tiab] OR "body mass index"[tiab:~0]))) AND (pool*[tiab] OR million[tiab] OR cohort*[tiab] OR risk*[ti] OR incidence[ti] OR associat*[ti] ) AND english[la] NOT (review[pt] OR "Case Reports"[pt] OR "Comment"[pt] OR "Health Education"[MeSH] OR "prediction model"[ti] OR surviv*[ti] OR patient*[ti] OR odds[tiab] OR "ORs"[tiab] ) NOT (Mendelian random*[ti] OR polymorphisms[ti] OR pathway*[ti] OR expression[ti] OR DNA[ti] OR microRNA[ti] OR mRNA[ti] OR polymorphism[ti] OR virus[ti]) AND 2014:2023[pdat] |
| Colorectum | (colorectal neoplasms [MeSH] OR "colorectal cancer" OR "colon cancer" OR "rectal cancer" OR "rectum cancer") AND ("body mass index"[ti] OR bmi[ti] OR ((obesity[ti] OR adipos*[ti] OR body fat*[ti] OR body weight[ti] OR body mass[ti] OR body composition[ti] OR lifestyle[ti] OR anthropom*[ti]) AND (bmi[tiab] OR "body mass index"[tiab:~0]))) AND (pool*[tiab] OR million[tiab] OR cohort*[tiab] OR risk*[ti] OR incidence[ti] OR associat*[ti] ) AND english[la] NOT (review[pt] OR "Case Reports"[pt] OR "Comment"[pt] OR "Health Education"[MeSH] OR "prediction model"[ti] OR surviv*[ti] OR patient*[ti] OR odds[tiab] OR "ORs"[tiab] ) NOT (Mendelian random*[ti] OR polymorphisms[ti] OR pathway*[ti] OR expression[ti] OR DNA[ti] OR microRNA[ti] OR mRNA[ti] OR polymorphism[ti] OR virus[ti]) AND 2014:2023[pdat] |
| Brain, central nervous system | ("brain neoplasms"[MeSH] OR "meningioma"[MeSH] OR "glioma"[MeSH] OR "brain cancer" OR "glioblastoma") AND ("body mass index"[ti] OR bmi[ti] OR ((obesity[ti] OR adipos*[ti] OR body fat*[ti] OR body weight[ti] OR body mass[ti] OR body composition[ti] OR lifestyle[ti] OR anthropom*[ti]) AND (bmi[tiab] OR "body mass index"[tiab:~0]))) AND (pool*[tiab] OR million[tiab] OR cohort*[tiab] OR risk*[ti] OR incidence[ti] OR associat*[ti] ) AND english[la] NOT (review[pt] OR "Case Reports"[pt] OR "Comment"[pt] OR "Health Education"[MeSH] OR "prediction model"[ti] OR surviv*[ti] OR patient*[ti] OR odds[tiab] OR "ORs"[tiab] ) NOT (Mendelian random*[ti] OR polymorphisms[ti] OR pathway*[ti] OR expression[ti] OR DNA[ti] OR microRNA[ti] OR mRNA[ti] OR polymorphism[ti] OR virus[ti]) AND 2013:2023[pdat] |
| Gallbladder | (gallbladder neoplasm OR "gallbladder cancer") AND ("body mass index"[ti] OR bmi[ti] OR ((obesity[ti] OR adipos*[ti] OR body fat*[ti] OR body weight[ti] OR body mass[ti] OR body composition[ti] OR lifestyle[ti] OR anthropom*[ti]) AND (bmi[tiab] OR "body mass index"[tiab:~0]))) AND (pool*[tiab] OR million[tiab] OR cohort*[tiab] OR risk*[ti] OR incidence[ti] OR associat*[ti] ) AND english[la] NOT (review[pt] OR "Case Reports"[pt] OR "Comment"[pt] OR "Health Education"[MeSH] OR "prediction model"[ti] OR surviv*[ti] OR patient*[ti] OR odds[tiab] OR "ORs"[tiab] ) NOT (Mendelian random*[ti] OR polymorphisms[ti] OR pathway*[ti] OR expression[ti] OR DNA[ti] OR microRNA[ti] OR mRNA[ti] OR polymorphism[ti] OR virus[ti]) AND 2013:2023[pdat] |
| [Oesophagus](file:///C:\Users\wattsel\AppData\Local\Microsoft\Windows\INetCache\Content.MSO\CEED827C.xlsx) | (esophageal neoplasms[MeSH] OR "oesophageal cancer" OR "esophageal cancer" OR esophag*[tiab] OR oesophag*[tiab]) AND ("body mass index"[ti] OR bmi[ti] OR ((obesity[ti] OR adipos*[ti] OR body fat*[ti] OR body weight[ti] OR body mass[ti] OR body composition[ti] OR lifestyle[ti] OR anthropom*[ti]) AND (bmi[tiab] OR "body mass index"[tiab:~0]))) AND (pool*[tiab] OR million[tiab] OR cohort*[tiab] OR risk*[ti] OR incidence[ti] OR associat*[ti] ) AND english[la] NOT (review[pt] OR "Case Reports"[pt] OR "Comment"[pt] OR "Health Education"[MeSH] OR "prediction model"[ti] OR surviv*[ti] OR patient*[ti] OR odds[tiab] OR "ORs"[tiab] ) NOT (Mendelian random*[ti] OR polymorphisms[ti] OR pathway*[ti] OR expression[ti] OR DNA[ti] OR microRNA[ti] OR mRNA[ti] OR polymorphism[ti] OR virus[ti]) AND 2013:2023[pdat] |
| Liver | (liver neoplasms [MeSH] OR "liver cancer" OR hepatocarcinoma[tiab]) AND ("body mass index"[ti] OR bmi[ti] OR ((obesity[ti] OR adipos*[ti] OR body fat*[ti] OR body weight[ti] OR body mass[ti] OR body composition[ti] OR lifestyle[ti] OR anthropom*[ti]) AND (bmi[tiab] OR "body mass index"[tiab:~0]))) AND (pool*[tiab] OR million[tiab] OR cohort*[tiab] OR risk*[ti] OR incidence[ti] OR associat*[ti] ) AND english[la] NOT (review[pt] OR "Case Reports"[pt] OR "Comment"[pt] OR "Health Education"[MeSH] OR "prediction model"[ti] OR surviv*[ti] OR patient*[ti] OR odds[tiab] OR "ORs"[tiab] ) NOT (Mendelian random*[ti] OR polymorphisms[ti] OR pathway*[ti] OR expression[ti] OR DNA[ti] OR microRNA[ti] OR mRNA[ti] OR polymorphism[ti] OR virus[ti]) AND 2012:2023[pdat] |
| Ovary | (ovarian neoplasms[MeSH] OR "ovarian cancer") AND ("body mass index"[ti] OR bmi[ti] OR ((obesity[ti] OR adipos*[ti] OR body fat*[ti] OR body weight[ti] OR body mass[ti] OR body composition[ti] OR lifestyle[ti] OR anthropom*[ti]) AND (bmi[tiab] OR "body mass index"[tiab:~0]))) AND (pool*[tiab] OR million[tiab] OR cohort*[tiab] OR risk*[ti] OR incidence[ti] OR associat*[ti] ) AND english[la] NOT (review[pt] OR "Case Reports"[pt] OR "Comment"[pt] OR "Health Education"[MeSH] OR "prediction model"[ti] OR surviv*[ti] OR patient*[ti] OR odds[tiab] OR "ORs"[tiab] ) NOT (Mendelian random*[ti] OR polymorphisms[ti] OR pathway*[ti] OR expression[ti] OR DNA[ti] OR microRNA[ti] OR mRNA[ti] OR polymorphism[ti] OR virus[ti]) AND 2012:2023[pdat] |
| Prostate | (prostatic neoplasms[MeSH] OR "prostate cancer") AND ("body mass index"[ti] OR bmi[ti] OR ((obesity[ti] OR adipos*[ti] OR body fat*[ti] OR body weight[ti] OR body mass[ti] OR body composition[ti] OR lifestyle[ti] OR anthropom*[ti]) AND (bmi[tiab] OR "body mass index"[tiab:~0]))) AND (pool*[tiab] OR million[tiab] OR cohort*[tiab] OR risk*[ti] OR incidence[ti] OR associat*[ti] ) AND english[la] NOT (review[pt] OR "Case Reports"[pt] OR "Comment"[pt] OR "Health Education"[MeSH] OR "prediction model"[ti] OR surviv*[ti] OR patient*[ti] OR odds[tiab] OR "ORs"[tiab] ) NOT (Mendelian random*[ti] OR polymorphisms[ti] OR pathway*[ti] OR expression[ti] OR DNA[ti] OR microRNA[ti] OR mRNA[ti] OR polymorphism[ti] OR virus[ti]) AND 2012:2023[pdat] |
| [Endometrium](file:///C:\Users\wattsel\AppData\Local\Microsoft\Windows\INetCache\Content.MSO\CEED827C.xlsx) | (endometrial neoplasm[MeSH] OR "endometrial cancer" OR "uterine corpus" OR "corpus uteri") AND ("body mass index"[ti] OR bmi[ti] OR ((obesity[ti] OR adipos*[ti] OR body fat*[ti] OR body weight[ti] OR body mass[ti] OR body composition[ti] OR lifestyle[ti] OR anthropom*[ti]) AND (bmi[tiab] OR "body mass index"[tiab:~0]))) AND (pool*[tiab] OR million[tiab] OR cohort*[tiab] OR risk*[ti] OR incidence[ti] OR associat*[ti] ) AND english[la] NOT (review[pt] OR "Case Reports"[pt] OR "Comment"[pt] OR "Health Education"[MeSH] OR "prediction model"[ti] OR surviv*[ti] OR patient*[ti] OR odds[tiab] OR "ORs"[tiab] ) NOT (Mendelian random*[ti] OR polymorphisms[ti] OR pathway*[ti] OR expression[ti] OR DNA[ti] OR microRNA[ti] OR mRNA[ti] OR polymorphism[ti] OR virus[ti]) AND 2011:2023[pdat] |
| Bladder | ("urinary bladder neoplasms"[MeSH Terms] OR "bladder cancer") AND ("body mass index"[ti] OR bmi[ti] OR ((obesity[ti] OR adipos*[ti] OR body fat*[ti] OR body weight[ti] OR body mass[ti] OR body composition[ti] OR lifestyle[ti] OR anthropom*[ti]) AND (bmi[tiab] OR "body mass index"[tiab:~0]))) AND (pool*[tiab] OR million[tiab] OR cohort*[tiab] OR risk*[ti] OR incidence[ti] OR associat*[ti] ) AND english[la] NOT (review[pt] OR "Case Reports"[pt] OR "Comment"[pt] OR "Health Education"[MeSH] OR "prediction model"[ti] OR surviv*[ti] OR patient*[ti] OR odds[tiab] OR "ORs"[tiab] ) NOT (Mendelian random*[ti] OR polymorphisms[ti] OR pathway*[ti] OR expression[ti] OR DNA[ti] OR microRNA[ti] OR mRNA[ti] OR polymorphism[ti] OR virus[ti]) AND 2010:2023[pdat] |
| Kidney | (kidney neoplasms [MeSH] OR "kidney cancer" OR "renal cancer") AND ("body mass index"[ti] OR bmi[ti] OR ((obesity[ti] OR adipos*[ti] OR body fat*[ti] OR body weight[ti] OR body mass[ti] OR body composition[ti] OR lifestyle[ti] OR anthropom*[ti]) AND (bmi[tiab] OR "body mass index"[tiab:~0]))) AND (pool*[tiab] OR million[tiab] OR cohort*[tiab] OR risk*[ti] OR incidence[ti] OR associat*[ti] ) AND english[la] NOT (review[pt] OR "Case Reports"[pt] OR "Comment"[pt] OR "Health Education"[MeSH] OR "prediction model"[ti] OR surviv*[ti] OR patient*[ti] OR odds[tiab] OR "ORs"[tiab] ) NOT (Mendelian random*[ti] OR polymorphisms[ti] OR pathway*[ti] OR expression[ti] OR DNA[ti] OR microRNA[ti] OR mRNA[ti] OR polymorphism[ti] OR virus[ti]) AND 2010:2023[pdat] |
| Pancreas | (pancreatic neoplasms[MeSH] OR "pancreatic cancer") AND ("body mass index"[ti] OR bmi[ti] OR ((obesity[ti] OR adipos*[ti] OR body fat*[ti] OR body weight[ti] OR body mass[ti] OR body composition[ti] OR lifestyle[ti] OR anthropom*[ti]) AND (bmi[tiab] OR "body mass index"[tiab:~0]))) AND (pool*[tiab] OR million[tiab] OR cohort*[tiab] OR risk*[ti] OR incidence[ti] OR associat*[ti] ) AND english[la] NOT (review[pt] OR "Case Reports"[pt] OR "Comment"[pt] OR "Health Education"[MeSH] OR "prediction model"[ti] OR surviv*[ti] OR patient*[ti] OR odds[tiab] OR "ORs"[tiab] ) NOT (Mendelian random*[ti] OR polymorphisms[ti] OR pathway*[ti] OR expression[ti] OR DNA[ti] OR microRNA[ti] OR mRNA[ti] OR polymorphism[ti] OR virus[ti]) AND 2010:2023[pdat] |
| Stomach (gastric) | (stomach neoplasms[MeSH] OR "stomach cancer" OR "gastric cancer" OR "gastric cardia") AND ("body mass index"[ti] OR bmi[ti] OR ((obesity[ti] OR adipos*[ti] OR body fat*[ti] OR body weight[ti] OR body mass[ti] OR body composition[ti] OR lifestyle[ti] OR anthropom*[ti]) AND (bmi[tiab] OR "body mass index"[tiab:~0]))) AND (pool*[tiab] OR million[tiab] OR cohort*[tiab] OR risk*[ti] OR incidence[ti] OR associat*[ti] ) AND english[la] NOT (review[pt] OR "Case Reports"[pt] OR "Comment"[pt] OR "Health Education"[MeSH] OR "prediction model"[ti] OR surviv*[ti] OR patient*[ti] OR odds[tiab] OR "ORs"[tiab] ) NOT (Mendelian random*[ti] OR polymorphisms[ti] OR pathway*[ti] OR expression[ti] OR DNA[ti] OR microRNA[ti] OR mRNA[ti] OR polymorphism[ti] OR virus[ti]) AND 2008:2023[pdat] |
| Lung | ("lung neoplasms"[MeSH Terms] OR "lung cancer") AND ("body mass index"[ti] OR bmi[ti] OR ((obesity[ti] OR adipos*[ti] OR body fat*[ti] OR body weight[ti] OR body mass[ti] OR body composition[ti] OR lifestyle[ti] OR anthropom*[ti]) AND (bmi[tiab] OR "body mass index"[tiab:~0]))) AND (pool*[tiab] OR million[tiab] OR cohort*[tiab] OR risk*[ti] OR incidence[ti] OR associat*[ti] ) AND english[la] NOT (review[pt] OR "Case Reports"[pt] OR "Comment"[pt] OR "Health Education"[MeSH] OR "prediction model"[ti] OR surviv*[ti] OR patient*[ti] OR odds[tiab] OR "ORs"[tiab] ) NOT (Mendelian random*[ti] OR polymorphisms[ti] OR pathway*[ti] OR expression[ti] OR DNA[ti] OR microRNA[ti] OR mRNA[ti] OR polymorphism[ti] OR virus[ti]) AND 2014:2023[pdat] |

| Site | Current meta-analysis |  |  |  |  |  | WCRF reviews |  |  |  |  |  | Meta-analysis vs. WCRF |  |
| --- | --- | --- | --- | --- | --- | --- | --- | --- | --- | --- | --- | --- | --- | --- |
|  | N cases | Analytic cohort | N cohorts | N countries | N regions | RR per 5 kg/m^2^ (95% CI) | N cases | N cohorts | N countries | N regions | Last study year | RR per 5 kg/m^2^ (95% CI) | Case N ratio | Change in RR |
| Endometrial | 44,449 | 15,761,666 | 38 | 17 | 3 | 1.59 (1.52, 1.67) | 18,941 | 25 | 15 | 3 | 2011 | 1.50 (1.42, 1.59) | 2:1 | 0.09 |
| Oesophageal (adeno) | 4,405 | 6,117,888 | 13 | 13 | 3 | 1.51 (1.41, 1.61) | 1,988 | 9 | 7 | 2 | 2013 | 1.48 (1.35, 1.62) | 2:1 | 0.03 |
| Kidney | 43,376 | 38,745,179 | 25 | 16 | 3 | 1.30 (1.26, 1.33) | 16,076 | 23 | 15 | 3 | 2010 | 1.30 (1.25, 1.35) | 3:1 | 0.00 |
| Liver | 67,828 | 27,512,631 | 29 | 16 | 3 | 1.26 (1.17, 1.36) | 11,664 | 8 | 13 | 2 | 2012 | 1.30 (1.16, 1.46) | 6:1 | -0.04 |
| Gallbladder | 10,649 | 16,627,205 | 38 | 19 | 4 | 1.22 (1.17, 1.28) | 5,745 | 8 | 13 | 3 | 2013 | 1.25 (1.15, 1.37) | 2:1 | -0.03 |
| Gastric (cardia) | 4,723 | 2,959,571 | 13 | 15 | 4 | 1.22 (1.14, 1.31) | 2,050 | 7 | 6 | 3 | 2008 | 1.23 (1.07, 1.40) | 2:1 | -0.01 |
| Meningioma | 1,380 | 2,535,565 | 7 | 13 | 3 | 1.22 (1.14, 1.32) | - | - | - | - | - | - | ∞ | - |
| Breast (postmenopausal) | 168,366 | 14,737,434 | 38 | 19 | 4 | 1.13 (1.11, 1.16) | 78,847 | 42 | 18 | 4 | 2015 | 1.12 (1.09, 1.15) | 2:1 | 0.01 |
| Head and neck (NS) | 10,841 | 11,257,993 | 25 | 15 | 4 | 1.12 (1.04, 1.21) | 796 | 20 | 13 | 4 | 2015 | 1.15 (1.06, 1.24) | 14:1 | -0.03 |
| Thyroid | 18,512 | 13,073,624 | 41 | 17 | 4 | 1.11 (1.05, 1.17) | 2,996 | 22 | 14 | 4 | 2016 | 1.06 (1.02, 1.10) | 6:1 | 0.05 |
| Colorectal | 268,307 | 39,938,241 | 48 | 22 | 5 | 1.10 (1.08, 1.12) | 46,359 | 29 | 13 | 4 | 2014 | 1.05 (1.03, 1.07) | 6:1 | 0.05 |
| Leukaemia | 19,928 | 14,111,203 | 16 | 16 | 3 | 1.09 (1.05, 1.13) | - | - | - | - | - | - | ∞ | - |
| Pancreas | 39,866 | 22,313,575 | 36 | 18 | 5 | 1.08 (1.06, 1.10) | 9,401 | 23 | 14 | 3 | 2010 | 1.10 (1.07, 1.14) | 4:1 | -0.02 |
| Multiple myeloma | 15,688 | 13,636,385 | 21 | 13 | 3 | 1.08 (1.05, 1.10) | 7,862 | 16 | 13 | 3 | 2014 | 1.06 (1.03, 1.10) | 2:1 | 0.02 |
| Ovarian | 32,071 | 14,075,874 | 29 | 17 | 4 | 1.07 (1.04, 1.10) | 15,773 | 22 | 16 | 4 | 2012 | 1.06 (1.02, 1.11) | 2:1 | 0.01 |
| Prostate (aggressive)* | 19,069 | 2,670,266 | 28 | 17 | 4 | 1.06 (1.02, 1.09) | 9,838 | 23 | 14 | 4 | 2012 | 1.08 (1.04, 1.12) | 2:1 | -0.02 |
| Bladder (NS) | 21,257 | 20,741,237 | 9 | 11 | 3 | 1.05 (1.02, 1.07) | - | - | - | - | - | - | ∞ | - |
| NHL | 35,338 | 15,674,504 | 21 | 10 | 3 | 1.05 (1.03, 1.07) | - | - | - | - | - | - | ∞ | - |
| Cervix | 11,018 | 11,507,843 | 12 | 15 | 3 | 1.04 (0.99, 1.10) | 5,035 | 9 | 7 | 3 | 2014 | 1.02 (0.97, 1.07) | 2:1 | 0.02 |
| Glioma | 6,840 | 9,791,602 | 9 | 14 | 3 | 1.03 (1.01, 1.05) | - | - | - | - | - | - | ∞ | - |
| Melanoma | 36,014 | 13,636,275 | 18 | 13 | 4 | 1.03 (0.98, 1.08) | 17,689 | 14 | 9 | 4 | 2016 | 1.02 (0.98-1.05) | 2:1 | 0.01 |
| Gastric (non-cardia) | 44,667 | 9,111,437 | 16 | 18 | 5 | 0.96 (0.92, 1.01) | 2,462 | 7 | 7 | 4 | 2008 | 0.93 (0.85, 1.02) | 18:1 | 0.03 |
| Lung (NS) | 10,893 | 7,020,028 | 13 | 17 | 4 | 0.96 (0.93, 0.99) | 4,712 | 12 | 7 | 4 | 2014 | 0.93 (0.84, 1.03) | 2:1 | 0.03 |
| Breast (premenopausal) | 52,457 | 12,313,546 | 43 | 19 | 4 | 0.91 (0.89, 0.93) | 16,371 | 27 | 14 | 3 | 2015 | 0.93 (0.90, 0.97) | 3:1 | -0.02 |
| Oesophageal (SQ, NS) | 893 | 1,857,723 | 4 | 11 | 2 | 0.77 (0.63, 0.94) | 114 | 2 | 9 | 1 | 2009 | 0.59 (0.44, 0.79) | 8:1 | 0.18 |

 Supplementary Table 3: Comparison of current meta-analysis results with WCRF reviews

*Aggressive prostate cancer was defined as any of stage 3-4 on the American Joint Committee on Cancer 1992 classification, advanced cancer, advanced or metastatic cancer; metastatic cancer; stage C or D on the Whitmore/Jewett scale; fatal cancer (prostate cancer-specific mortality); high stage or grade; Gleason grade ≥ 7). 

Abbreviations: NS=never-smokers, SQ=squamous cell carcinoma, RR=risk ratio, WCRF=World Cancer Research Fund

Supplementary Table 4: Pooled estimates with and without large health databases included

|  | Meta-analysis |  |  | Large health databases excluded |  |  |  |
| --- | --- | --- | --- | --- | --- | --- | --- |
| Site | Case N | RR per 5 kg/m^2 ^(95% CI) |  | Case N | RR per 5 kg/m^2 ^(95% CI) |  | Change in RRs |
| Endometrial | 44,449 | 1.59 (1.52, 1.67) |  | 29,779 | 1.60 (1.52, 1.68) |  | -0.01 |
| Oesophageal (adeno) | 4,405 | 1.51 (1.41, 1.61) |  | 4,405 | 1.51 (1.41, 1.61) |  | - |
| Kidney | 43,376 | 1.30 (1.26, 1.33) |  | 17,310 | 1.31 (1.28, 1.34) |  | -0.01 |
| Liver | 67,828 | 1.26 (1.17, 1.36) |  | 13,050 | 1.29 (1.19, 1.40) |  | -0.03 |
| Gastric (cardia) | 4,723 | 1.22 (1.14, 1.31) |  | 4,723 | 1.22 (1.14, 1.31) |  | - |
| Gallbladder | 10,649 | 1.22 (1.17, 1.28) |  | 7,402 | 1.26 (1.21, 1.31) |  | -0.04 |
| Meningioma | 1,380 | 1.22 (1.14, 1.32) |  | 1,380 | 1.22 (1.14, 1.32) |  | - |
| Breast (postmenopausal) | 168,366 | 1.13 (1.10, 1.16) |  | 81,477 | 1.13 (1.10, 1.16) |  | 0.00 |
| Head and neck (NS) | 10,045 | 1.12 (1.04, 1.21) |  | 271 | 1.17 (1.07, 1.28) |  | -0.05 |
| Thyroid | 18,507 | 1.11 (1.05, 1.17) |  | 14,878 | 1.11 (1.04, 1.17) |  | 0.00 |
| Colorectal | 268,307 | 1.10 (1.08, 1.12) |  | 97,254 | 1.10 (1.08, 1.12) |  | 0.00 |
| Pancreas | 39,971 | 1.08 (1.06, 1.10) |  | 18,909 | 1.10 (1.08, 1.12) |  | -0.02 |
| Leukaemia | 19,928 | 1.09 (1.05, 1.13) |  | 6,960 | 1.09 (1.04, 1.13) |  | 0.00 |
| Multiple myeloma | 15,688 | 1.08 (1.06, 1.10) |  | 9,249 | 1.08 (1.05, 1.11) |  | 0.00 |
| Ovarian | 32,071 | 1.07 (1.04, 1.10) |  | 18,848 | 1.06 (1.03, 1.09) |  | 0.01 |
| Prostate (aggressive)* | 19,069 | 1.06 (1.02, 1.09) |  | 18,780 | 1.04 (1.02, 1.07) |  | 0.02 |
| Bladder (NS) | 21,257 | 1.05 (1.02, 1.07) |  | 2,548 | 1.05 (0.99, 1.11) |  | 0.00 |
| Non-Hodgkin lymphoma | 35,338 | 1.05 (1.03, 1.07) |  | 21,405 | 1.06 (1.03, 1.08) |  | -0.01 |
| Cervix | 11,018 | 1.03 (0.99, 1.08) |  | 1,974 | 1.02 (0.95, 1.09) |  | 0.01 |
| Glioma | 6,840 | 1.06 (1.02, 1.09) |  | 2,369 | 1.04 (0.98, 1.09) |  | 0.02 |
| Melanoma | 36,014 | 1.03 (0.98, 1.08) |  | 20,624 | 1.03 (0.98, 1.08) |  | 0.00 |
| Lung (NS) | 10,893 | 0.96 (0.93, 0.99) |  | 4,663 | 0.95 (0.90, 1.00) |  | 0.01 |
| Gastric (non-cardia) | 44,667 | 0.96 (0.92, 1.01) |  | 14,892 | 0.97 (0.93, 1.02) |  | -0.01 |
| Breast (premenopausal) | 58,834 | 0.91 (0.89, 0.93) |  | 33,744 | 0.91 (0.89, 0.93) |  | 0.00 |
| Oesophageal (SQ, NS) | 893 | 0.77 (0.63, 0.94) |  | 893 | 0.77 (0.63, 0.94) |  | - |

Risk estimates were combined using weighted averages of the log RRs in the separate studies, with weights assigned based on within and between study variance, yielding a pooled risk estimate and its 95% CI. Pooled estimates are displayed with and without inclusion of 3 large national/regional healthcare databases (KNHIS, SIDIAP, CPRD)^36,38,39^.

*Aggressive prostate cancer was defined as any of stage 3-4 on the American Joint Committee on Cancer 1992 classification, advanced cancer, advanced or metastatic cancer; metastatic cancer; stage C or D on the Whitmore/Jewett scale; fatal cancer (prostate cancer-specific mortality); high stage or grade; Gleason grade ≥ 7). 

Abbreviations: Adeno=adenocarcinoma, CI=confidence interval, CPRD=UK Clinical Practice Research Datalink, KNHIS=National Health Insurance Service of Korea, NS=never-smokers, PMID=PubMed ID, RR=risk ratio, SIDIAP=Information System for Research in Primary Care, SQ=squamous cell carcinoma.

**Supplementary Figures**

Studies identified (N total=2,425—includes duplicates across cancer types):

WCRF/meta-analysis (N=522)

PubMed (N=1,632)

PubMed returned for other cancer site or reference search (N=271)

Exclusions:

Non-cancer incidence outcome (N=1,183)

N<50,000 cohort participants (N=81)

Not convertible to continuous estimate (N=23)

No risk estimates available for subtypes (breast, prostate, gastric, CNS, oesophageal) (N=67)

No risk estimates for never-smokers (head and neck, lung, oesophageal (SQ) and bladder cancers) (N=36)

Did not evaluate confounding by smoking (N=43)

HRs adjusted for adiposity measurements (N=6)

Duplicate cohort with fewer cases (N=319)

Duplicate study (N=63)

Included:

BMI-cancer risk estimates (N=688)

Distinct PMIDs (N=250)

 

Supplementary Figure 1: Studies identified by search strategy*

*Some publications investigated multiple cancer sites and therefore results do not sum. Exclusion numbers for each type of cancer are available from **Supplementary Table 1**. 

Abbreviations: CNS=central nervous system, HR=hazard ratio, PMID=PubMed ID, SQ=squamous cell carcinoma, WCRF=World Cancer Research Fund

**A**

**B**

Supplementary Figure 2: Percentage of cancer cases originating from each geographic region

**A: Sum of the 25 types of incident cancers** 

**B: Individual cancers**

Pooled estimates spanning multiple regions for which country-specific case numbers were not available were excluded (postmenopausal breast, lung and head and neck)^35–37^. 

 

Supplementary Figure 3: Meta-analysis of prospective studies on the risk of endometrial cancer in relation to BMI

Study-specific RRs are represented by squares (with their 95% CIs as lines). RRs were combined using weighted averages of the log RRs in the separate studies, with weights assigned based on within and between study variance, yielding a pooled risk estimate and its 95% CI (diamond). Heterogeneity across studies was assessed using I^2^, tau^2^, and the 25th and 75th percentile of the RRs. Further details of model adjustments, follow-up time, analytic population for each study are available from **Supplementary Data 1**.

Abbreviations: BMI=body mass index, CI=confidence interval, PMID=PubMed ID, RR=risk ratio

Supplementary Figure 4: Meta-analysis of prospective studies on the risk of oesophageal adenocarcinoma cancer in relation to BMI

Study-specific RRs are represented by squares (with their 95% CIs as lines). RRs were combined using weighted averages of the log RRs in the separate studies, with weights assigned based on within and between study variance, yielding a pooled risk estimate and its 95% CI (diamond). Heterogeneity across studies was assessed using I^2^, tau^2^, and the 25th and 75th percentile of the RRs. Further details of model adjustments, follow-up time, analytic population for each study are available from **Supplementary Data 1**.

Abbreviations: BMI=body mass index, CI=confidence interval, PMID=PubMed ID, RR=risk ratio

Supplementary Figure 5: Meta-analysis of prospective studies on the risk of kidney cancer in relation to BMI

Study-specific RRs are represented by squares (with their 95% CIs as lines). RRs were combined using weighted averages of the log RRs in the separate studies, with weights assigned based on within and between study variance, yielding a pooled risk estimate and its 95% CI (diamond). Heterogeneity across studies was assessed using I^2^, tau^2^, and the 25th and 75th percentile of the RRs. Further details of model adjustments, follow-up time, analytic population for each study are available from **Supplementary Data 1**.

Abbreviations: BMI=body mass index, CI=confidence interval, PMID=PubMed ID, RR=risk ratio

 

Supplementary Figure 6: Meta-analysis of prospective studies on the risk of liver cancer in relation to BMI

Study-specific RRs are represented by squares (with their 95% CIs as lines). RRs were combined using weighted averages of the log RRs in the separate studies, with weights assigned based on within and between study variance, yielding a pooled risk estimate and its 95% CI (diamond). Heterogeneity across studies was assessed using I^2^, tau^2^, and the 25th and 75th percentile of the RRs. Further details of model adjustments, follow-up time, analytic population for each study are available from **Supplementary Data 1**.

Abbreviations: BMI=body mass index, CI=confidence interval, PMID=PubMed ID, RR=risk ratio

Supplementary Figure 7: Meta-analysis of prospective studies on the risk of gastric cancer (cardia) in relation to BMI

Study-specific RRs are represented by squares (with their 95% CIs as lines). RRs were combined using weighted averages of the log RRs in the separate studies, with weights assigned based on within and between study variance, yielding a pooled risk estimate and its 95% CI (diamond). Heterogeneity across studies was assessed using I^2^, tau^2^, and the 25th and 75th percentile of the RRs. Further details of model adjustments, follow-up time, analytic population for each study are available from **Supplementary Data 1**.

Abbreviations: BMI=body mass index, CI=confidence interval, PMID=PubMed ID, RR=risk ratio

Supplementary Figure 8: Meta-analysis of prospective studies on the risk of gallbladder cancer in relation to BMI

Study-specific RRs are represented by squares (with their 95% CIs as lines). RRs were combined using weighted averages of the log RRs in the separate studies, with weights assigned based on within and between study variance, yielding a pooled risk estimate and its 95% CI (diamond). Heterogeneity across studies was assessed using I^2^, tau^2^, and the 25th and 75th percentile of the RRs. Further details of model adjustments, follow-up time, analytic population for each study are available from **Supplementary Data 1**.

Abbreviations: BMI=body mass index, CI=confidence interval, PMID=PubMed ID, RR=risk ratio

Supplementary Figure 9: Meta-analysis of prospective studies on the risk of meningioma in relation to BMI

Study-specific RRs are represented by squares (with their 95% CIs as lines). RRs were combined using weighted averages of the log RRs in the separate studies, with weights assigned based on within and between study variance, yielding a pooled risk estimate and its 95% CI (diamond). Heterogeneity across studies was assessed using I^2^, tau^2^, and the 25th and 75th percentile of the RRs. Further details of model adjustments, follow-up time, analytic population for each study are available from **Supplementary Data 1**.

Abbreviations: BMI=body mass index, CI=confidence interval, PMID=PubMed ID, RR=risk ratio

Supplementary Figure 10: Meta-analysis of prospective studies on the risk of breast (postmenopausal) cancer in relation to BMI

Study-specific RRs are represented by squares (with their 95% CIs as lines). RRs were combined using weighted averages of the log RRs in the separate studies, with weights assigned based on within and between study variance, yielding a pooled risk estimate and its 95% CI (diamond). Heterogeneity across studies was assessed using I^2^, tau^2^, and the 25th and 75th percentile of the RRs. Further details of model adjustments, follow-up time, analytic population for each study are available from **Supplementary Data 1**.

Abbreviations: BMI=body mass index, CI=confidence interval, PMID=PubMed ID, RR=risk ratio

Supplementary Figure 11: Meta-analysis of prospective studies on the risk of head and neck cancer (restricted to never-smokers) in relation to BMI

Study-specific RRs are represented by squares (with their 95% CIs as lines). RRs were combined using weighted averages of the log RRs in the separate studies, with weights assigned based on within and between study variance, yielding a pooled risk estimate and its 95% CI (diamond). Heterogeneity across studies was assessed using I^2^, tau^2^, and the 25th and 75th percentile of the RRs. Further details of model adjustments, follow-up time, analytic population for each study are available from **Supplementary Data 1**. Cases of head and neck cancers for never-smokers were not available in individual studies (N cases total=796).

Abbreviations: BMI=body mass index, CI=confidence interval, PMID=PubMed ID, RR=risk ratio

Supplementary Figure 12: Meta-analysis of prospective studies on the risk of thyroid cancer in relation to BMI

Study-specific RRs are represented by squares (with their 95% CIs as lines). RRs were combined using weighted averages of the log RRs in the separate studies, with weights assigned based on within and between study variance, yielding a pooled risk estimate and its 95% CI (diamond). Heterogeneity across studies was assessed using I^2^, tau^2^, and the 25th and 75th percentile of the RRs. Further details of model adjustments, follow-up time, analytic population for each study are available from **Supplementary Data 1**.

Abbreviations: BMI=body mass index, CI=confidence interval, PMID=PubMed ID, RR=risk ratio

Figure 13: Meta-analysis of prospective studies on the risk of colorectal cancer in relation to BMI

Study-specific RRs are represented by squares (with their 95% CIs as lines). RRs were combined using weighted averages of the log RRs in the separate studies, with weights assigned based on within and between study variance, yielding a pooled risk estimate and its 95% CI (diamond). Heterogeneity across studies was assessed using I^2^, tau^2^, and the 25th and 75th percentile of the RRs. Further details of model adjustments, follow-up time, analytic population for each study are available from **Supplementary Data 1**.

Abbreviations: BMI=body mass index, CI=confidence interval, PMID=PubMed ID, RR=risk ratio

Supplementary Figure 14: Meta-analysis of prospective studies on the risk of pancreatic cancer in relation to BMI

Study-specific RRs are represented by squares (with their 95% CIs as lines). RRs were combined using weighted averages of the log RRs in the separate studies, with weights assigned based on within and between study variance, yielding a pooled risk estimate and its 95% CI (diamond). Heterogeneity across studies was assessed using I^2^, tau^2^, and the 25th and 75th percentile of the RRs. Further details of model adjustments, follow-up time, analytic population for each study are available from **Supplementary Data 1**.

Abbreviations: BMI=body mass index, CI=confidence interval, PMID=PubMed ID, RR=risk ratio

Supplementary Figure 15: Meta-analysis of prospective studies on the risk of leukaemia in relation to BMI

Study-specific RRs are represented by squares (with their 95% CIs as lines). RRs were combined using weighted averages of the log RRs in the separate studies, with weights assigned based on within and between study variance, yielding a pooled risk estimate and its 95% CI (diamond). Heterogeneity across studies was assessed using I^2^, tau^2^, and the 25th and 75th percentile of the RRs. Further details of model adjustments, follow-up time, analytic population for each study are available from **Supplementary Data 1**.

Abbreviations: BMI=body mass index, CI=confidence interval, PMID=PubMed ID, RR=risk ratio

Supplementary Figure 16: Meta-analysis of prospective studies on the risk of multiple myeloma in relation to BMI

Study-specific RRs are represented by squares (with their 95% CIs as lines). RRs were combined using weighted averages of the log RRs in the separate studies, with weights assigned based on within and between study variance, yielding a pooled risk estimate and its 95% CI (diamond). Heterogeneity across studies was assessed using I^2^, tau^2^, and the 25th and 75th percentile of the RRs. Further details of model adjustments, follow-up time, analytic population for each study are available from **Supplementary Data 1**.

Abbreviations: BMI=body mass index, CI=confidence interval, PMID=PubMed ID, RR=risk ratio

Supplementary Figure 17: Meta-analysis of prospective studies on the risk of ovarian cancer in relation to BMI

Study-specific RRs are represented by squares (with their 95% CIs as lines). RRs were combined using weighted averages of the log RRs in the separate studies, with weights assigned based on within and between study variance, yielding a pooled risk estimate and its 95% CI (diamond). Heterogeneity across studies was assessed using I^2^, tau^2^, and the 25th and 75th percentile of the RRs. Further details of model adjustments, follow-up time, analytic population for each study are available from **Supplementary Data 1**.

Abbreviations: BMI=body mass index, CI=confidence interval, PMID=PubMed ID, RR=risk ratio

Supplementary Figure 18: Meta-analysis of prospective studies on the risk of prostate cancer (aggressive) in relation to BMI

Aggressive prostate cancer was defined as any of stage 3-4 on the American Joint Committee on Cancer 1992 classification, advanced cancer, advanced or metastatic cancer; metastatic cancer; stage C or D on the Whitmore/Jewett scale; fatal cancer (prostate cancer-specific mortality); high stage or grade; Gleason grade ≥ 7). Study-specific RRs are represented by squares (with their 95% CIs as lines). RRs were combined using weighted averages of the log RRs in the separate studies, with weights assigned based on within and between study variance, yielding a pooled risk estimate and its 95% CI (diamond). Heterogeneity across studies was assessed using I^2^, tau^2^, and the 25th and 75th percentile of the RRs. Further details of model adjustments, follow-up time, analytic population for each study are available from **Supplementary Data 1**. 

Abbreviations: BMI=body mass index, CI=confidence interval, PMID=PubMed ID, RR=risk ratio

Supplementary Figure 19: Meta-analysis of prospective studies on the risk of bladder cancer (restricted to never-smokers) in relation to BMI

Study-specific RRs are represented by squares (with their 95% CIs as lines). RRs were combined using weighted averages of the log RRs in the separate studies, with weights assigned based on within and between study variance, yielding a pooled risk estimate and its 95% CI (diamond). Heterogeneity across studies was assessed using I^2^, tau^2^, and the 25th and 75th percentile of the RRs. Further details of model adjustments, follow-up time, analytic population for each study are available from **Supplementary Data 1**.

Abbreviations: BMI=body mass index, CI=confidence interval, PMID=PubMed ID, RR=risk ratio

Supplementary Figure 20: Meta-analysis of prospective studies on the risk of NHL in relation to BMI

Study-specific RRs are represented by squares (with their 95% CIs as lines). RRs were combined using weighted averages of the log RRs in the separate studies, with weights assigned based on within and between study variance, yielding a pooled risk estimate and its 95% CI (diamond). Heterogeneity across studies was assessed using I^2^, tau^2^, and the 25th and 75th percentile of the RRs. Further details of model adjustments, follow-up time, analytic population for each study are available from **Supplementary Data 1**.

Abbreviations: BMI=body mass index, CI=confidence interval, NHL=non-Hodgkin lymphoma, PMID=PubMed ID, RR=risk ratio

Supplementary Figure 21: Meta-analysis of prospective studies on the risk of cervical cancer in relation to BMI

Study-specific RRs are represented by squares (with their 95% CIs as lines). RRs were combined using weighted averages of the log RRs in the separate studies, with weights assigned based on within and between study variance, yielding a pooled risk estimate and its 95% CI (diamond). Heterogeneity across studies was assessed using I^2^, tau^2^, and the 25th and 75th percentile of the RRs. Further details of model adjustments, follow-up time, analytic population for each study are available from **Supplementary Data 1**.

Abbreviations: BMI=body mass index, CI=confidence interval, PMID=PubMed ID, RR=risk ratio

Supplementary Figure 22: Meta-analysis of prospective studies on the risk of glioma in relation to BMI

Study-specific RRs are represented by squares (with their 95% CIs as lines). RRs were combined using weighted averages of the log RRs in the separate studies, with weights assigned based on within and between study variance, yielding a pooled risk estimate and its 95% CI (diamond). Heterogeneity across studies was assessed using I^2^, tau^2^, and the 25th and 75th percentile of the RRs. Further details of model adjustments, follow-up time, analytic population for each study are available from **Supplementary Data 1**.

Abbreviations: BMI=body mass index, CI=confidence interval, PMID=PubMed ID, RR=risk ratio

Supplementary Figure 23: Meta-analysis of prospective studies on the risk of melanoma in relation to BMI

Study-specific RRs are represented by squares (with their 95% CIs as lines). RRs were combined using weighted averages of the log RRs in the separate studies, with weights assigned based on within and between study variance, yielding a pooled risk estimate and its 95% CI (diamond). Heterogeneity across studies was assessed using I^2^, tau^2^, and the 25th and 75th percentile of the RRs. Further details of model adjustments, follow-up time, analytic population for each study are available from **Supplementary Data 1**.

Abbreviations: BMI=body mass index, CI=confidence interval, PMID=PubMed ID, RR=risk ratio

Supplementary Figure 24: Meta-analysis of prospective studies on the risk of lung cancer (restricted to never-smokers) in relation to BMI

Study-specific RRs are represented by squares (with their 95% CIs as lines). RRs were combined using weighted averages of the log RRs in the separate studies, with weights assigned based on within and between study variance, yielding a pooled risk estimate and its 95% CI (diamond). Heterogeneity across studies was assessed using I^2^, tau^2^, and the 25th and 75th percentile of the RRs. Further details of model adjustments, follow-up time, analytic population for each study are available from **Supplementary Data 1**.

Abbreviations: BMI=body mass index, CI=confidence interval, PMID=PubMed ID, RR=risk ratio

Supplementary Figure 25: Meta-analysis of prospective studies on the risk of gastric cancer (non-cardia) in relation to BMI

Study-specific RRs are represented by squares (with their 95% CIs as lines). RRs were combined using weighted averages of the log RRs in the separate studies, with weights assigned based on within and between study variance, yielding a pooled risk estimate and its 95% CI (diamond). Heterogeneity across studies was assessed using I^2^, tau^2^, and the 25th and 75th percentile of the RRs. Further details of model adjustments, follow-up time, analytic population for each study are available from **Supplementary Data 1**.

Abbreviations: BMI=body mass index, CI=confidence interval, PMID=PubMed ID, RR=risk ratio

Supplementary Figure 26: Meta-analysis of prospective studies on the risk of breast cancer (premenopausal) in relation to BMI

Study-specific RRs are represented by squares (with their 95% CIs as lines). RRs were combined using weighted averages of the log RRs in the separate studies, with weights assigned based on within and between study variance, yielding a pooled risk estimate and its 95% CI (diamond). Heterogeneity across studies was assessed using I^2^, tau^2^, and the 25th and 75th percentile of the RRs. Further details of model adjustments, follow-up time, analytic population for each study are available from **Supplementary Data 1**.

Abbreviations: BMI=body mass index, CI=confidence interval, PMID=PubMed ID, RR=risk ratio

Supplementary Figure 27: Meta-analysis of prospective studies on the risk of oesophageal (SQ, restricted to never-smokers) in relation to BMI

Study-specific RRs are represented by squares (with their 95% CIs as lines). RRs were combined using weighted averages of the log RRs in the separate studies, with weights assigned based on within and between study variance, yielding a pooled risk estimate and its 95% CI (diamond). Heterogeneity across studies was assessed using I^2^, tau^2^, and the 25th and 75th percentile of the RRs. Further details of model adjustments, follow-up time, analytic population for each study are available from **Supplementary Data 1**.

Abbreviations: BMI=body mass index, CI=confidence interval, PMID=PubMed ID, RR=risk ratio, SQ=squamous cell carcinoma. 

Supplementary Figure 28: Meta-analysis of prospective studies on the risk of endometrial cancer in relation to BMI, by region

Study-specific RRs are represented by squares (with their 95% CIs as lines). RRs were combined using random effects meta-analysis (represented by a diamond). Heterogeneity across studies was assessed using I^2^ and tau^2^, within region and across all regions. Heterogeneity in the pooled estimates by region was assessed using the Q-statistic. Further details of model adjustments, follow-up time, analytic population for each study are available from **Supplementary Data 1**.

Abbreviations: BMI=body mass index, CI=confidence interval, PMID=PubMed ID, RR=risk ratio

Supplementary Figure 29: Meta-analysis of prospective studies on the risk of oesophageal adenocarcinoma cancer in relation to BMI, by region

Study-specific RRs are represented by squares (with their 95% CIs as lines). RRs were combined using random effects meta-analysis (represented by a diamond). Heterogeneity across studies was assessed using I^2^ and tau^2^, within region and across all regions. Heterogeneity in the pooled estimates by region was assessed using the Q-statistic. Further details of model adjustments, follow-up time, analytic population for each study are available from **Supplementary Data 1**.

Abbreviations: BMI=body mass index, CI=confidence interval, PMID=PubMed ID, RR=risk ratio

Supplementary Figure 30: Meta-analysis of prospective studies on the risk of kidney cancer in relation to BMI, by region

Study-specific RRs are represented by squares (with their 95% CIs as lines). RRs were combined using random effects meta-analysis (represented by a diamond). Heterogeneity across studies was assessed using I^2^ and tau^2^, within region and across all regions. Heterogeneity in the pooled estimates by region was assessed using the Q-statistic. Further details of model adjustments, follow-up time, analytic population for each study are available from **Supplementary Data 1**.

Abbreviations: BMI=body mass index, CI=confidence interval, PMID=PubMed ID, RR=risk ratio

Supplementary Figure 31: Meta-analysis of prospective studies on the risk of liver cancer in relation to BMI, by region

Study-specific RRs are represented by squares (with their 95% CIs as lines). RRs were combined using random effects meta-analysis (represented by a diamond). Heterogeneity across studies was assessed using I^2^ and tau^2^, within region and across all regions. Heterogeneity in the pooled estimates by region was assessed using the Q-statistic. Further details of model adjustments, follow-up time, analytic population for each study are available from **Supplementary Data 1**.

Abbreviations: BMI=body mass index, CI=confidence interval, PMID=PubMed ID, RR=risk ratio

Supplementary Figure 32: Meta-analysis of prospective studies on the risk of gastric cancer (cardia) in relation to BMI, by region

Study-specific RRs are represented by squares (with their 95% CIs as lines). RRs were combined using random effects meta-analysis (represented by a diamond). Heterogeneity across studies was assessed using I^2^ and tau^2^, within region and across all regions. Heterogeneity in the pooled estimates by region was assessed using the Q-statistic. Further details of model adjustments, follow-up time, analytic population for each study are available from **Supplementary Data 1**.

Abbreviations: BMI=body mass index, CI=confidence interval, PMID=PubMed ID, RR=risk ratio

Supplementary Figure 33: Meta-analysis of prospective studies on the risk of gallbladder cancer in relation to BMI, by region

Study-specific RRs are represented by squares (with their 95% CIs as lines). RRs were combined using random effects meta-analysis (represented by a diamond). Heterogeneity across studies was assessed using I^2^ and tau^2^, within region and across all regions. Heterogeneity in the pooled estimates by region was assessed using the Q-statistic. Further details of model adjustments, follow-up time, analytic population for each study are available from **Supplementary Data 1**.

Abbreviations: BMI=body mass index, CI=confidence interval, PMID=PubMed ID, RR=risk ratio

Supplementary Figure 34: Meta-analysis of prospective studies on the risk of meningioma cancer in relation to BMI, by region

Study-specific RRs are represented by squares (with their 95% CIs as lines). RRs were combined using random effects meta-analysis (represented by a diamond). Heterogeneity across studies was assessed using I^2^ and tau^2^, within region and across all regions. Heterogeneity in the pooled estimates by region was assessed using the Q-statistic. Further details of model adjustments, follow-up time, analytic population for each study are available from **Supplementary Data 1**.

Abbreviations: BMI=body mass index, CI=confidence interval, PMID=PubMed ID, RR=risk ratio

Figure 35: Meta-analysis of prospective studies on the risk of breast cancer (postmenopausal) in relation to BMI, by region 

Study-specific RRs are represented by squares (with their 95% CIs as lines). RRs were combined using random effects meta-analysis (represented by a diamond). Heterogeneity across studies was assessed using I^2^ and tau^2^, within region and across all regions. Heterogeneity in the pooled estimates by region was assessed using the Q-statistic. Further details of model adjustments, follow-up time, analytic population for each study are available from **Supplementary Data 1**. Pooled estimates spanning multiple regions where region-specific RRs were not available were excluded^29^.

Abbreviations: BMI=body mass index, CI=confidence interval, PMID=PubMed ID, RR=risk ratio

Supplementary Figure 36: Meta-analysis of prospective studies on the risk of head and neck cancer (restricted to never-smokers) in relation to BMI, by region

 Study-specific RRs are represented by squares (with their 95% CIs as lines). RRs were combined using random effects meta-analysis (represented by a diamond). Heterogeneity across studies was assessed using I^2^ and tau^2^, within region and across all regions. Heterogeneity in the pooled estimates by region was assessed using the Q-statistic. Further details of model adjustments, follow-up time, analytic population for each study are available from **Supplementary Data 1**. Cases numbers for never-smokers for each individual study were not specified in Gaudet et al., 2015 (total cases=796).

Abbreviations: BMI=body mass index, CI=confidence interval, PMID=PubMed ID, RR=risk ratio

Supplementary Figure 37: Meta-analysis of prospective studies on the risk of thyroid cancer in relation to BMI, by region

Study-specific RRs are represented by squares (with their 95% CIs as lines). RRs were combined using random effects meta-analysis (represented by a diamond). Heterogeneity across studies was assessed using I^2^ and tau^2^, within region and across all regions. Heterogeneity in the pooled estimates by region was assessed using the Q-statistic. Further details of model adjustments, follow-up time, analytic population for each study are available from **Supplementary Data 1**.

Abbreviations: BMI=body mass index, CI=confidence interval, PMID=PubMed ID, RR=risk ratio

Supplementary Figure 38: Meta-analysis of prospective studies on the risk of colorectal cancer in relation to BMI, by region

Study-specific RRs are represented by squares (with their 95% CIs as lines). RRs were combined using random effects meta-analysis (represented by a diamond). Heterogeneity across studies was assessed using I^2^ and tau^2^, within region and across all regions. Heterogeneity in the pooled estimates by region was assessed using the Q-statistic. Further details of model adjustments, follow-up time, analytic population for each study are available from **Supplementary Data 1**.

Abbreviations: BMI=body mass index, CI=confidence interval, PMID=PubMed ID, RR=risk ratio

Supplementary Figure 39: Meta-analysis of prospective studies on the risk of pancreatic cancer in relation to BMI, by region

Study-specific RRs are represented by squares (with their 95% CIs as lines). RRs were combined using random effects meta-analysis (represented by a diamond). Heterogeneity across studies was assessed using I^2^ and tau^2^, within region and across all regions. Heterogeneity in the pooled estimates by region was assessed using the Q-statistic. Further details of model adjustments, follow-up time, analytic population for each study are available from **Supplementary Data 1**.

Abbreviations: BMI=body mass index, CI=confidence interval, PMID=PubMed ID, RR=risk ratio

Supplementary Figure 40: Meta-analysis of prospective studies on the risk of leukaemia in relation to BMI, by region

Study-specific RRs are represented by squares (with their 95% CIs as lines). RRs were combined using random effects meta-analysis (represented by a diamond). Heterogeneity across studies was assessed using I^2^ and tau^2^, within region and across all regions. Heterogeneity in the pooled estimates by region was assessed using the Q-statistic. Further details of model adjustments, follow-up time, analytic population for each study are available from **Supplementary Data 1**.

Abbreviations: BMI=body mass index, CI=confidence interval, PMID=PubMed ID, RR=risk ratio

Supplementary Figure 41: Meta-analysis of prospective studies on the risk of multiple myeloma in relation to BMI, by region

Study-specific RRs are represented by squares (with their 95% CIs as lines). RRs were combined using random effects meta-analysis (represented by a diamond). Heterogeneity across studies was assessed using I^2^ and tau^2^, within region and across all regions. Heterogeneity in the pooled estimates by region was assessed using the Q-statistic. Further details of model adjustments, follow-up time, analytic population for each study are available from **Supplementary Data 1**.

Abbreviations: BMI=body mass index, CI=confidence interval, PMID=PubMed ID, RR=risk ratio

Supplementary Figure 42: Meta-analysis of prospective studies on the risk of ovarian cancer in relation to BMI, by region

Study-specific RRs are represented by squares (with their 95% CIs as lines). RRs were combined using random effects meta-analysis (represented by a diamond). Heterogeneity across studies was assessed using I^2^ and tau^2^, within region and across all regions. Heterogeneity in the pooled estimates by region was assessed using the Q-statistic. Further details of model adjustments, follow-up time, analytic population for each study are available from **Supplementary Data 1**.

Abbreviations: BMI=body mass index, CI=confidence interval, PMID=PubMed ID, RR=risk ratio

Supplementary Figure 43: Meta-analysis of prospective studies on the risk of aggressive prostate cancer in relation to BMI, by region

Aggressive prostate cancer was defined as any of stage 3-4 on the American Joint Committee on Cancer 1992 classification, advanced cancer, advanced or metastatic cancer; metastatic cancer; stage C or D on the Whitmore/Jewett scale; fatal cancer (prostate cancer-specific mortality); high stage or grade; Gleason grade ≥ 7). Study-specific RRs are represented by squares (with their 95% CIs as lines). RRs were combined using random effects meta-analysis (represented by a diamond). Heterogeneity across studies was assessed using I^2^ and tau^2^, within region and across all regions. Heterogeneity in the pooled estimates by region was assessed using the Q-statistic. Further details of model adjustments, follow-up time, analytic population for each study are available from **Supplementary Data 1**.

Abbreviations: BMI=body mass index, CI=confidence interval, PMID=PubMed ID, RR=risk ratio

Supplementary Figure 44: Meta-analysis of prospective studies on the risk of bladder cancer (restricted to never-smokers) in relation to BMI, by region

Study-specific RRs are represented by squares (with their 95% CIs as lines). RRs were combined using random effects meta-analysis (represented by a diamond). Heterogeneity across studies was assessed using I^2^ and tau^2^, within region and across all regions. Heterogeneity in the pooled estimates by region was assessed using the Q-statistic. Further details of model adjustments, follow-up time, analytic population for each study are available from **Supplementary Data 1**.

Abbreviations: BMI=body mass index, CI=confidence interval, PMID=PubMed ID, RR=risk ratio

Supplementary Figure 45: Meta-analysis of prospective studies on the risk of NHL in relation to BMI, by region

Study-specific RRs are represented by squares (with their 95% CIs as lines). RRs were combined using random effects meta-analysis (represented by a diamond). Heterogeneity across studies was assessed using I^2^ and tau^2^, within region and across all regions. Heterogeneity in the pooled estimates by region was assessed using the Q-statistic. Further details of model adjustments, follow-up time, analytic population for each study are available from **Supplementary Data 1**.

Abbreviations: BMI=body mass index, CI=confidence interval, NHL=non-Hodgkin lymphoma, PMID=PubMed ID, RR=risk ratio

Supplementary Figure 46: Meta-analysis of prospective studies on the risk of cervical cancer in relation to BMI, by region

Study-specific RRs are represented by squares (with their 95% CIs as lines). RRs were combined using random effects meta-analysis (represented by a diamond). Heterogeneity across studies was assessed using I^2^ and tau^2^, within region and across all regions. Heterogeneity in the pooled estimates by region was assessed using the Q-statistic. Further details of model adjustments, follow-up time, analytic population for each study are available from **Supplementary Data 1**.

Abbreviations: BMI=body mass index, CI=confidence interval, PMID=PubMed ID, RR=risk ratio

Supplementary Figure 47: Meta-analysis of prospective studies on the risk of glioma in relation to BMI, by region

Study-specific RRs are represented by squares (with their 95% CIs as lines). RRs were combined using random effects meta-analysis (represented by a diamond). Heterogeneity across studies was assessed using I^2^ and tau^2^, within region and across all regions. Heterogeneity in the pooled estimates by region was assessed using the Q-statistic. Further details of model adjustments, follow-up time, analytic population for each study are available from **Supplementary Data 1**.

Abbreviations: BMI=body mass index, CI=confidence interval, PMID=PubMed ID, RR=risk ratio

Supplementary Figure 48: Meta-analysis of prospective studies on the risk of melanoma in relation to BMI, by region

Study-specific RRs are represented by squares (with their 95% CIs as lines). RRs were combined using random effects meta-analysis (represented by a diamond). Heterogeneity across studies was assessed using I^2^ and tau^2^, within region and across all regions. Heterogeneity in the pooled estimates by region was assessed using the Q-statistic. Further details of model adjustments, follow-up time, analytic population for each study are available from **Supplementary Data 1**.

Abbreviations: BMI=body mass index, CI=confidence interval, PMID=PubMed ID, RR=risk ratio

Supplementary Figure 49: Meta-analysis of prospective studies on the risk of lung cancer (restricted to never-smokers) in relation to BMI, by region

Study-specific RRs are represented by squares (with their 95% CIs as lines). RRs were combined using random effects meta-analysis (represented by a diamond). Heterogeneity across studies was assessed using I^2^ and tau^2^, within region and across all regions. Heterogeneity in the pooled estimates by region was assessed using the Q-statistic. Further details of model adjustments, follow-up time, analytic population for each study are available from **Supplementary Data 1**.

Abbreviations: BMI=body mass index, CI=confidence interval, PMID=PubMed ID, RR=risk ratio

Supplementary Figure 50: Meta-analysis of prospective studies on the risk of gastric cancer (non-cardia) in relation to BMI, by region 

Study-specific RRs are represented by squares (with their 95% CIs as lines). RRs were combined using random effects meta-analysis (represented by a diamond). Heterogeneity across studies was assessed using I^2^ and tau^2^, within region and across all regions. Heterogeneity in the pooled estimates by region was assessed using the Q-statistic. Further details of model adjustments, follow-up time, analytic population for each study are available from **Supplementary Data 1**.

Abbreviations: BMI=body mass index, CI=confidence interval, PMID=PubMed ID, RR=risk ratio

Supplementary Figure 51: Meta-analysis of prospective studies on the risk of breast cancer (premenopausal) in relation to BMI, by region

 Study-specific RRs are represented by squares (with their 95% CIs as lines). RRs were combined using random effects meta-analysis (represented by a diamond). Heterogeneity across studies was assessed using I^2^ and tau^2^, within region and across all regions. Heterogeneity in the pooled estimates by region was assessed using the Q-statistic. Further details of model adjustments, follow-up time, analytic population for each study are available from **Supplementary Data 1**.

Abbreviations: BMI=body mass index, CI=confidence interval, PMID=PubMed ID, RR=risk ratio

Supplementary Figure 52: Meta-analysis of prospective studies on the risk of oesophageal SQ in relation to BMI, by region

Study-specific RRs are represented by squares (with their 95% CIs as lines). RRs were combined using random effects meta-analysis (represented by a diamond). Heterogeneity across studies was assessed using I^2^ and tau^2^, within region and across all regions. Heterogeneity in the pooled estimates by region was assessed using the Q-statistic. Further details of model adjustments, follow-up time, analytic population for each study are available from **Supplementary Data 1**.

Abbreviations: BMI=body mass index, CI=confidence interval, PMID=PubMed ID, RR=risk ratio, SQ=squamous cell carcinoma. 

Supplementary Figure 53: Associations of BMI, waist circumference and endometrial cancer risk, per 1 SD increase

Study-specific RRs are represented by squares (with their 95% CIs as lines). RRs were combined using random effects meta-analysis (represented by a diamond). Heterogeneity across studies was assessed using I^2^ and tau^2^, within adiposity measure. Heterogeneity in the pooled estimates by adiposity measurement was assessed using the Wald statistic.  

Abbreviations: BMI=body mass index, CI=confidence interval, PMID=PubMed ID, RR=risk ratio, SD=standard deviation, WC=waist circumference.

Supplementary Figure 54: Associations of BMI, waist circumference and oesophageal adenocarcinoma risk, per 1 SD increase

Study-specific RRs are represented by squares (with their 95% CIs as lines). RRs were combined using random effects meta-analysis (represented by a diamond). Heterogeneity across studies was assessed using I^2^ and tau^2^, within adiposity measure. Heterogeneity in the pooled estimates by adiposity measurement was assessed using the Wald statistic.

Abbreviations: BMI=body mass index, CI=confidence interval, PMID=PubMed ID, RR=risk ratio, SD=standard deviation, WC=waist circumference.

Supplementary Figure 55: Associations of BMI, waist circumference and liver cancer risk, per 1 SD increase

Study-specific RRs are represented by squares (with their 95% CIs as lines). RRs were combined using random effects meta-analysis (represented by a diamond). Heterogeneity across studies was assessed using I^2^ and tau^2^, within adiposity measure. Heterogeneity in the pooled estimates by adiposity measurement was assessed using the Wald statistic.  

Abbreviations: BMI=Body mass index, CI=confidence interval, PMID=PubMed ID, RR=risk ratio, SD=standard deviation, WC=waist circumference.

Supplementary Figure 56: Associations of BMI, waist circumference and kidney cancer risk, per 1 SD increase

Study-specific RRs are represented by squares (with their 95% CIs as lines). RRs were combined using random effects meta-analysis (represented by a diamond). Heterogeneity across studies was assessed using I^2^ and tau^2^, within adiposity measure. Heterogeneity in the pooled estimates by adiposity measurement was assessed using the Wald statistic.  

Abbreviations: BMI=Body mass index, CI=confidence interval, PMID=PubMed ID, RR=risk ratio, SD=standard deviation, WC=waist circumference.

Supplementary Figure 57: Associations of BMI, waist circumference and gallbladder cancer risk, per 1 SD increase

Study-specific RRs are represented by squares (with their 95% CIs as lines). RRs were combined using random effects meta-analysis (represented by a diamond). Heterogeneity across studies was assessed using I^2^ and tau^2^, within adiposity measure. Heterogeneity in the pooled estimates by adiposity measurement was assessed using the Wald statistic.  

Abbreviations: BMI=Body mass index, CI=confidence interval, PMID=PubMed ID, RR=risk ratio, SD=standard deviation, WC=waist circumference.

Supplementary Figure 58: Associations of BMI, waist circumference and gastric cancer (cardia) risk, per 1 SD increase

Study-specific RRs are represented by squares (with their 95% CIs as lines). RRs were combined using random effects meta-analysis (represented by a diamond). Heterogeneity across studies was assessed using I^2^ and tau^2^, within adiposity measure. Heterogeneity in the pooled estimates by adiposity measurement was assessed using the Wald statistic.  

Abbreviations: BMI=Body mass index, CI=confidence interval, PMID=PubMed ID, RR=risk ratio, SD=standard deviation, WC=waist circumference.

Supplementary Figure 59: Associations of BMI, waist circumference and breast (postmenopausal) cancer risk, per 1 SD increase

Study-specific RRs are represented by squares (with their 95% CIs as lines). RRs were combined using random effects meta-analysis (represented by a diamond). Heterogeneity across studies was assessed using I^2^ and tau^2^, within adiposity measure. Heterogeneity in the pooled estimates by adiposity measurement was assessed using the Wald statistic.  

Abbreviations: BMI=Body mass index, CI=confidence interval, PMID=PubMed ID, RR=risk ratio, SD=standard deviation, WC=waist circumference.

Supplementary Figure 60: Associations of BMI, waist circumference and pancreatic cancer risk, per 1 SD increase

Study-specific RRs are represented by squares (with their 95% CIs as lines). RRs were combined using random effects meta-analysis (represented by a diamond). Heterogeneity across studies was assessed using I^2^ and tau^2^, within adiposity measure. Heterogeneity in the pooled estimates by adiposity measurement was assessed using the Wald statistic.  

Abbreviations: BMI=Body mass index, CI=confidence interval, PMID=PubMed ID, RR=risk ratio, SD=standard deviation, WC=waist circumference.

Supplementary Figure 61: Associations of BMI, waist circumference and head and neck cancer risk, per 1 SD increase

Study-specific RRs are represented by squares (with their 95% CIs as lines). RRs were combined using random effects meta-analysis (represented by a diamond). Heterogeneity across studies was assessed using I^2^ and tau^2^, within adiposity measure. Heterogeneity in the pooled estimates by adiposity measurement was assessed using the Wald statistic.  

Abbreviations: BMI=Body mass index, CI=confidence interval, PMID=PubMed ID, RR=risk ratio, SD=standard deviation, WC=waist circumference.

Supplementary Figure 62: Associations of BMI, waist circumference and thyroid cancer risk, per 1 SD increase

Study-specific RRs are represented by squares (with their 95% CIs as lines). RRs were combined using random effects meta-analysis (represented by a diamond). Heterogeneity across studies was assessed using I^2^ and tau^2^, within adiposity measure. Heterogeneity in the pooled estimates by adiposity measurement was assessed using the Wald statistic.

Abbreviations: BMI=Body mass index, CI=confidence interval, PMID=PubMed ID, RR=risk ratio, SD=standard deviation, WC=waist circumference.

Supplementary Figure 63: Associations of BMI, waist circumference and colorectal cancer risk, per 1 SD increase

Study-specific RRs are represented by squares (with their 95% CIs as lines). RRs were combined using random effects meta-analysis (represented by a diamond). Heterogeneity across studies was assessed using I^2^ and tau^2^, within adiposity measure. Heterogeneity in the pooled estimates by adiposity measurement was assessed using the Wald statistic.

Abbreviations: BMI=Body mass index, CI=confidence interval, PMID=PubMed ID, RR=risk ratio, SD=standard deviation, WC=waist circumference.

Supplementary Figure 64: Associations of BMI, waist circumference and leukaemia risk, per 1 SD increase

Study-specific RRs are represented by squares (with their 95% CIs as lines). RRs were combined using random effects meta-analysis (represented by a diamond). Heterogeneity across studies was assessed using I^2^ and tau^2^, within adiposity measure. Heterogeneity in the pooled estimates by adiposity measurement was assessed using the Wald statistic.  

Abbreviations: BMI=Body mass index, CI=confidence interval, PMID=PubMed ID, RR=risk ratio, SD=standard deviation, WC=waist circumference.

Supplementary Figure 65: Associations of BMI, waist circumference and multiple myeloma risk, per 1 SD increase

Study-specific RRs are represented by squares (with their 95% CIs as lines). RRs were combined using random effects meta-analysis (represented by a diamond). Heterogeneity across studies was assessed using I^2^ and tau^2^, within adiposity measure. Heterogeneity in the pooled estimates by adiposity measurement was assessed using the Wald statistic.  

Abbreviations: BMI=Body mass index, CI=confidence interval, PMID=PubMed ID, RR=risk ratio, SD=standard deviation, WC=waist circumference.

Supplementary Figure 66: Associations of BMI, waist circumference and ovarian cancer risk, per 1 SD increase

Study-specific RRs are represented by squares (with their 95% CIs as lines). RRs were combined using random effects meta-analysis (represented by a diamond). Heterogeneity across studies was assessed using I^2^ and tau^2^, within adiposity measure. Heterogeneity in the pooled estimates by adiposity measurement was assessed using the Wald statistic.

Abbreviations: BMI=Body mass index, CI=confidence interval, PMID=PubMed ID, RR=risk ratio, SD=standard deviation, WC=waist circumference.

Supplementary Figure 67: Associations of BMI, waist circumference and NHL risk, per 1 SD increase

Study-specific RRs are represented by squares (with their 95% CIs as lines). RRs were combined using random effects meta-analysis (represented by a diamond). Heterogeneity across studies was assessed using I^2^ and tau^2^, within adiposity measure. Heterogeneity in the pooled estimates by adiposity measurement was assessed using the Wald statistic.

Abbreviations: BMI=Body mass index, CI=confidence interval, NHL=non-Hodgkin lymphoma, PMID=PubMed ID, RR=risk ratio, SD=standard deviation, WC=waist circumference.

Supplementary Figure 68: Associations of BMI, waist circumference and melanoma risk, per 1 SD increase

Study-specific RRs are represented by squares (with their 95% CIs as lines). RRs were combined using random effects meta-analysis (represented by a diamond). Heterogeneity across studies was assessed using I^2^ and tau^2^, within adiposity measure. Heterogeneity in the pooled estimates by adiposity measurement was assessed using the Wald statistic.  

Abbreviations: BMI=Body mass index, CI=confidence interval, PMID=PubMed ID, RR=risk ratio, SD=standard deviation, WC=waist circumference.

Supplementary Figure 69: Associations of BMI, waist circumference and bladder cancer risk, per 1 SD increase

Study-specific RRs are represented by squares (with their 95% CIs as lines). RRs were combined using random effects meta-analysis (represented by a diamond). Heterogeneity across studies was assessed using I^2^ and tau^2^, within adiposity measure. Heterogeneity in the pooled estimates by adiposity measurement was assessed using the Wald statistic.

Abbreviations: BMI=Body mass index, CI=confidence interval, PMID=PubMed ID, RR=risk ratio, SD=standard deviation, WC=waist circumference.

Supplementary Figure 70: Associations of BMI, waist circumference and prostate cancer risk, per 1 SD increase

Study-specific RRs are represented by squares (with their 95% CIs as lines). RRs were combined using random effects meta-analysis (represented by a diamond). Heterogeneity across studies was assessed using I^2^ and tau^2^, within adiposity measure. Heterogeneity in the pooled estimates by adiposity measurement was assessed using the Wald statistic. 

Abbreviations: BMI=Body mass index, CI=confidence interval, PMID=PubMed ID, RR=risk ratio, SD=standard deviation, WC=waist circumference.

Supplementary Figure 71: Associations of BMI, waist circumference and cervical cancer risk, per 1 SD increase

Study-specific RRs are represented by squares (with their 95% CIs as lines). RRs were combined using random effects meta-analysis (represented by a diamond). Heterogeneity across studies was assessed using I^2^ and tau^2^, within adiposity measure. Heterogeneity in the pooled estimates by adiposity measurement was assessed using the Wald statistic.   

Abbreviations: BMI=Body mass index, CI=confidence interval, PMID=PubMed ID, RR=risk ratio, SD=standard deviation, WC=waist circumference.

Supplementary Figure 72: Associations of BMI, waist circumference and gastric cancer (non-cardia) risk, per 1 SD increase

Study-specific RRs are represented by squares (with their 95% CIs as lines). RRs were combined using random effects meta-analysis (represented by a diamond). Heterogeneity across studies was assessed using I^2^ and tau^2^, within adiposity measure. Heterogeneity in the pooled estimates by adiposity measurement was assessed using the Wald statistic.

Abbreviations: BMI=Body mass index, CI=confidence interval, PMID=PubMed ID, RR=risk ratio, SD=standard deviation, WC=waist circumference.

Supplementary Figure 73: Associations of BMI, waist circumference and lung cancer risk, per 1 SD increase

Study-specific RRs are represented by squares (with their 95% CIs as lines). RRs were combined using random effects meta-analysis (represented by a diamond). Heterogeneity across studies was assessed using I^2^ and tau^2^, within adiposity measure. Heterogeneity in the pooled estimates by adiposity measurement was assessed using the Wald statistic. 

Abbreviations: BMI=Body mass index, CI=confidence interval, PMID=PubMed ID, RR=risk ratio, SD=standard deviation, WC=waist circumference.

Supplementary Figure 74: Associations of BMI, waist circumference and lung cancer risk (restricted to never-smokers), per 1 SD increase

Study-specific RRs are represented by squares (with their 95% CIs as lines). RRs were combined using random effects meta-analysis (represented by a diamond). Heterogeneity across studies was assessed using I^2^ and tau^2^, within adiposity measure. Heterogeneity in the pooled estimates by adiposity measurement was assessed using the Wald statistic.

Abbreviations: BMI=Body mass index, CI=confidence interval, PMID=PubMed ID, RR=risk ratio, SD=standard deviation, WC=waist circumference.

Supplementary Figure 75: Associations of BMI, waist circumference and breast (premenopausal) cancer risk, per 1 SD increase

Study-specific RRs are represented by squares (with their 95% CIs as lines). RRs were combined using random effects meta-analysis (represented by a diamond). Heterogeneity across studies was assessed using I^2^ and tau^2^, within adiposity measure. Heterogeneity in the pooled estimates by adiposity measurement was assessed using the Wald statistic.

Abbreviations: BMI=Body mass index, CI=confidence interval, PMID=PubMed ID, RR=risk ratio, SD=standard deviation, WC=waist circumference.

Supplementary Figure 76: Associations of BMI, waist circumference and oesophageal SQ cancer risk, per 1 SD increase

Study-specific RRs are represented by squares (with their 95% CIs as lines). RRs were combined using random effects meta-analysis (represented by a diamond). Heterogeneity across studies was assessed using I^2^ and tau^2^, within adiposity measure. Heterogeneity in the pooled estimates by adiposity measurement was assessed using the Wald statistic. 

Abbreviations: BMI=Body mass index, CI=confidence interval, PMID=PubMed ID, RR=risk ratio, SD=standard deviation, SQ=squamous cell carcinoma, WC=waist circumference.

## 11. **References**

1.	Freedman, N. D. *et al.* Impact of changing US cigarette smoking patterns on incident cancer: risks of 20 smoking-related cancers among the women and men of the NIH-AARP cohort. *Int. J. Epidemiol.* **45**, 846–856 (2016).

2.	Tangen, C. M. *et al.* Biases in Recommendations for and Acceptance of Prostate Biopsy Significantly Affect Assessment of Prostate Cancer Risk Factors: Results From Two Large Randomized Clinical Trials. *J. Clin. Oncol.* **34**, 4338–4344 (2016).

3.	WCRF/AICR. Diet, nutrition, physical activity and prostate cancer. (2018).

4.	WCRF/AICR. Diet, nutrition, physical activity and cancers of the mouth, pharynx and larynx. (2018).

5.	WCRF/AICR. Diet, nutrition, physical activity and oesophageal cancer. (2018).

6.	WCRF/AICR. Diet, nutrition, physical activity and endometrial cancer. (2018).

7.	WCRF/AICR. Diet, nutrition, physical activity and liver cancer. (2018).

8.	WCRF/AICR. Diet, nutrition, physical activity and kidney cancer. (2018).

9.	WCRF/AICR. Diet, nutrition, physical activity and gallbladder cancer. (2018).

10.	WCRF/AICR. Diet, nutrition, physical activity and stomach cancer. (2018).

11.	WCRF/AICR. Diet, nutrition, physical activity and breast cancer. (2018).

12.	WCRF/AICR. Diet, nutrition, physical activity and pancreatic cancer. (2018).

13.	WCRF/AICR. Diet, nutrition, physical activity and ovarian cancer. (2018).

14.	WCRF/AICR. Diet, nutrition, physical activity and colorectal cancer. (2018).

15.	WCRF/AICR. Diet, nutrition, physical activity and cervical cancer. (2018).

16.	WCRF/AICR. Diet, nutrition, physical activity and bladder cancer. (2018).

17.	WCRF/AICR. Diet, nutrition, physical activity and skin cancer. (2019).

18.	WCRF/AICR. Diet, nutrition, physical activity and lung cancer. (2018).

19.	Abar, L. *et al.* Body size and obesity during adulthood, and risk of lympho-haematopoietic cancers: an update of the WCRF-AICR systematic review of published prospective studies. *Ann. Oncol. Off. J. Eur. Soc. Med. Oncol.* **30**, 528–541 (2019).

20.	Zhang, D. *et al.* Body mass index and risk of brain tumors: a systematic review and dose-response meta-analysis. *Eur. J. Clin. Nutr.* **70**, 757–765 (2016).

21.	Kitahara, C. M. *et al.* Anthropometric Factors and Thyroid Cancer Risk by Histological Subtype: Pooled Analysis of 22 Prospective Studies. *Thyroid Off. J. Am. Thyroid Assoc.* **26**, 306–318 (2016).

22.	Arnold, M., Ferlay, J., Henegouwen, M. I. van B. & Soerjomataram, I. Global burden of oesophageal and gastric cancer by histology and subsite in 2018. *Gut* **69**, 1564–1571 (2020).

23.	Larsson, S. C., Spyrou, N. & Mantzoros, C. S. Body fatness associations with cancer: evidence from recent epidemiological studies and future directions. *Metabolism* **137**, 155326 (2022).

24.	Yang, T. Y. O. *et al.* Postmenopausal endometrial cancer risk and body size in early life and middle age: prospective cohort study. *Br. J. Cancer* **107**, 169–175 (2012).

25.	Harvey, S. V. *et al.* Associations of life course obesity with endometrial cancer in the Epidemiology of Endometrial Cancer Consortium (E2C2). *Int. J. Epidemiol.* **52**, 1086–1099 (2023).

26.	Burón Pust, A. *et al.* Heterogeneity of colorectal cancer risk by tumour characteristics: Large prospective study of UK women. *Int. J. Cancer* **140**, 1082–1090 (2017).

27.	Genkinger, J. M. *et al.* A pooled analysis of 14 cohort studies of anthropometric factors and pancreatic cancer risk. *Int. J. Cancer* **129**, 1708–1717 (2011).

28.	Genkinger, J. M. *et al.* Measures of body fatness and height in early and mid-to-late adulthood and prostate cancer: risk and mortality in The Pooling Project of Prospective Studies of Diet and Cancer. *Ann. Oncol. Off. J. Eur. Soc. Med. Oncol.* **31**, 103–114 (2020).

29.	van den Brandt, P. A. *et al.* Body size and weight change over adulthood and risk of breast cancer by menopausal and hormone receptor status: a pooled analysis of 20 prospective cohort studies. *Eur. J. Epidemiol.* **36**, 37–55 (2021).

30.	Greenland, S. & Longnecker, M. P. Methods for trend estimation from summarized dose-response data, with applications to meta-analysis. *Am. J. Epidemiol.* **135**, 1301–1309 (1992).

31.	Crippa, A. & Orsini, N. Multivariate Dose-Response Meta-Analysis: The **dosresmeta** *R* Package. *J. Stat. Softw.* **72**, (2016).

32.	Schwarzer, G., Carpenter, J. R. & Rücker, G. *Meta-Analysis with R*. (Springer International Publishing, Cham, 2015). doi:10.1007/978-3-319-21416-0.

33.	Higgins, J. P. T. Commentary: Heterogeneity in meta-analysis should be expected and appropriately quantified. *Int. J. Epidemiol.* **37**, 1158–1160 (2008).

34.	Paule, R. C. & Mandel, J. Consensus Values and Weighting Factors. *J. Res. Natl. Bur. Stand. 1977* **87**, 377–385 (1982).

35.	Christakoudi, S., Tsilidis, K. K., Evangelou, E. & Riboli, E. Association of body-shape phenotypes with imaging measures of body composition in the UK Biobank cohort: relevance to colon cancer risk. *BMC Cancer* **21**, 1106 (2021).

36.	Bhaskaran, K. *et al.* Body-mass index and risk of 22 specific cancers: a population-based cohort study of 5·24 million UK adults. *The Lancet* **384**, 755–765 (2014).

37.	Arhi, C. S. *et al.* Comparison of cancer diagnosis recording between the Clinical Practice Research Datalink, Cancer Registry and Hospital Episodes Statistics. *Cancer Epidemiol.* **57**, 148–157 (2018).

38.	Kim, D. B. *et al.* Waist circumference, body mass index, and colorectal cancer risk according to diabetes status: A Korean nationwide population-based cohort study. *J. Gastroenterol. Hepatol.* **36**, 397–405 (2021).

39.	Recalde, M. *et al.* Longitudinal body mass index and cancer risk: a cohort study of 2.6 million Catalan adults. *Nat. Commun.* **14**, 3816 (2023).
